# High‐Refractive‐Index Chip with Periodically Fine‐Tuning Gratings for Tunable Virtual‐Wavevector Spatial Frequency Shift Universal Super‐Resolution Imaging

**DOI:** 10.1002/advs.202103835

**Published:** 2022-01-27

**Authors:** Mingwei Tang, Yubing Han, Dehao Ye, Qianwei Zhang, Chenlei Pang, Xiaowei Liu, Weidong Shen, Yaoguang Ma, Clemens F. Kaminski, Xu Liu, Qing Yang

**Affiliations:** ^1^ State Key Laboratory of Modern Optical Instrumentation College of Optical Science and Engineering International Research Center for Advanced Photonics Zhejiang University Hangzhou 310027 China; ^2^ Research Center for Humanoid Sensing Zhejiang Lab Hangzhou 311100 China; ^3^ Department of Chemical Engineering and Biotechnology University of Cambridge Cambridge CB30AS UK; ^4^ Collaborative Innovation Center of Extreme Optics Shanxi University Taiyuan 030006 China

**Keywords:** field of view, label‐free, super‐resolution chips, tunable virtual‐wavevector spatial frequency shift

## Abstract

Continued research in fields such as materials science and biomedicine requires the development of a super‐resolution imaging technique with a large field of view (FOV) and deep subwavelength resolution that is compatible with both fluorescent and nonfluorescent samples. Existing on‐chip super‐resolution methods exclusively focus on either fluorescent or nonfluorescent imaging, and, as such, there is an urgent requirement for a more general technique that is capable of both modes of imaging. In this study, to realize labeled and label‐free super‐resolution imaging on a single scalable photonic chip, a universal super‐resolution imaging method based on the tunable virtual‐wavevector spatial frequency shift (TVSFS) principle is introduced. Using this principle, imaging resolution can be improved more than threefold over the diffraction limit of a linear optical system. Here, diffractive units are fabricated on the chip's surface to provide wavevector‐variable evanescent wave illumination, enabling tunable spatial frequency shifts in the Fourier space. A large FOV and resolutions of *λ*/4.7 and *λ*/7.1 were achieved for label‐free and fluorescently labeled samples using a gallium phosphide (GaP) chip. With its large FOV, compatibility with different imaging modes, and monolithic integration, the proposed TVSFS chip may advance fields such as cell engineering, precision industry inspection, and chemical research.

## Graphical abstract

A universal super‐resolution microscopy is developed based on tunable virtual‐wavevector spatial frequency shift that performs imaging on a single photonic chip, at a resolution three times better than the Abbe diffraction limit. Large field of view (FOV) imaging and resolutions of *λ*/4.7 for label‐free samples and *λ*/7.1 for fluorescently labeled samples with this chip are achieved. The micromodule can realize fast, large‐FOV, and deep subwavelength resolution imaging on an ordinary microscope when installed.

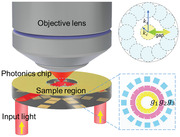

## 1 Introduction

The spatial resolution of conventional microscopy is limited to one‐half of the light wavelength by optical diffraction. The advent of fluorescence‐based super‐resolution microscopy has enabled the development of far‐field detection methods capable of discerning nanometer‐scale details, such as stimulated emission depletion microscopy (STED),^[^ ^1^, ^2^ ^]^ single‐molecule localization microscopy (SMLM),^[^ ^3^ ^]^ and minimal photon fluxes (MINFLUX).^[^ ^4^, ^5^ ^]^ With these methods, the diffraction limit is overcome in the spatial domain, by shrinking the point spread function (PSF) of the optical system. Although spatial resolutions down to 30 nm can easily be achieved with the aforementioned methods, they always require special fluorescent labeling.

The diffraction limit arises from the existence of information with high spatial frequency within the near‐field region of a specimen's surface, which cannot be detected by a far‐field objective. The near‐field information can be transformed into far‐field information using near‐field illumination techniques for detection, corresponding to the spatial frequency shift (SFS) in the frequency domain.^[^ ^6^, ^7^ ^]^ Compared with point scanning methods,^[^ ^1^, ^2^ ^]^ SFS methods can provide high‐speed, high‐resolution, wide‐field imaging. More importantly, in contrast with STED and SMLM methods, which rely on fluorescence excitation with specific characteristics, manipulation in the Fourier domain is universal and compatible with both label‐free and labeled imaging.

Examples of conventional SFS microscopy include structured illumination microscopy (SIM)^[^ ^8^, ^9^ ^]^ for labeled imaging and Fourier ptychographic microscopy (FPM)^[^ ^10^, ^11^ ^]^ for label‐free imaging. As both techniques use real‐wavevector free‐space light for illumination, the extension of the spatial frequency in the Fourier space is limited by the refractive index of the immersion medium used in imaging. The development of integrated waveguide and metamaterial paves the way for generating large lateral‐wavevector evanescent waves. By adopting high‐refractive‐index materials such as Al_2_O_3_,^[^ ^12^ ^]^ TiO_2_,^[^ ^13^ ^]^ Si_3_N_4_,^[^ ^14^, ^15^, ^16^, ^17^ ^]^ or dielectric/metal surface plasmon materials,^[^ ^18^, ^19^ ^]^ the resolution of SFS imaging techniques can reach subwavelength scales. However, existing SFS imaging methods meet two limitations. On the one hand, they operate exclusively as label‐free^[^ ^12^, ^13^, ^15^, ^16^ ^]^ or labeled imaging techniques^[^ ^14^, ^20^ ^]^ owing to the lack of a universal wavevector tuning method, or a flexible scheme for switching between the two imaging modes. On the other hand, there is a trade‐off between the lateral wavevector and the light propagation loss of a super‐resolution chip.^[^ ^18^, ^19^, ^21^ ^]^ Although metallic structures that generate surface plasmons can provide illumination with a large lateral wavevector, this can be accompanied by a non‐negligible ohmic loss,^[^ ^22^ ^]^ thus decreasing the continuous FOV. Conversely, although waveguides made from dielectric materials such as Al_2_O_3_ can propagate light over longer distances than are possible with metallic plasmons, they only provide a limited lateral wavevector.

**Figure 1:**
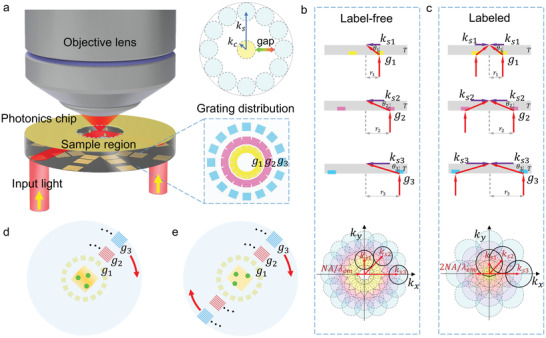
Physical scheme of chip‐based TVSFS super‐resolution imaging. a) Schematic illustration of chip‐based TVSFS super‐resolution imaging. Top right: Gap in the Fourier space obtained with high‐refractive‐index materials, explaining the necessity for multilevel tuning for SFS imaging. *k*_c_ and *k*_s_ are the cutoff and SFS wavevectors. b, c) Illustration of TVSFS imaging in the Fourier space and the principle of SFS tunability. d) Single‐beam illumination for label‐free imaging. e) Double‐beam illumination for labeled imaging.

In this paper, we propose a chip‐based universal tunable virtual‐wavevector spatial frequency shift (TVSFS) method, for both label‐free and labeled imaging, with which the resolution limit can be reduced considerably. The proposed TVSFS super‐resolution imaging is implemented on a CMOS‐compatible gallium phosphide (GaP) photonic chip, which has almost the highest refractive index (>3) in the visible spectrum. The dilemma of lateral wavevector and light propagation loss were circumvented by using a deflection scheme instead of surface propagation schemes. We fabricated micro/nanostructures on the surface of a transparent GaP substrate^[^ ^23^, ^24^ ^]^ (**Figure** **1**a), to generate surface waves on the other side of the substrate. The micro/nanostructures fabricated on the chip surface are designed to control the wavevector direction and amplitudes precisely to realize multilevel tuning, finally for wide coverage of the Fourier spectrum. The surface waves generated experience no optical loss, and can thus provide a large lateral wavevector and large FOV, determined respectively by the period and size of the fabricated micro/nanostructures, simultaneously. Besides, the chip‐based implementation makes the super‐resolution imaging simplified and maintenance costs reduced.^[^ ^14^, ^25^, ^26^, ^27^ ^]^

## 2 Results

For deep subwavelength imaging resolution, the magnitude of the illumination wavevector should be large. However, when the SFS magnitude (*k*_s_, which equals the magnitude of illumination wavevector divided by 2*π*) is more than double the radius of the maximal detection range of the collection objective in the frequency domain (*k*_c_), a gap is observed in the Fourier space (top‐right corner of Figure 1a), leading to artifacts in the final image, and sometimes even causing image reconstruction to fail.^[^ ^20^ ^]^ This gap can be filled through wavevector tuning, using either wavelength tuning for label‐free imaging,^[^ ^13^ ^]^ or multiangle tuning^[^ ^14^, ^20^ ^]^ for labeled imaging. To date, no tuning methods compatible with both labeled and label‐free samples, or capable of producing a tunable SFS vector with magnitude more than $\frac{2}{\lambda }$ for label‐free imaging and $\frac{4}{\lambda }$ for labeled imaging, have been reported. In this study, we develop a deep wavevector tuning method (with SFS magnitude tuning range beyond the limit of $\frac{2}{\lambda }$ for label‐free imaging and $\frac{4}{\lambda }$ for labeled imaging) that is compatible with both fluorescent and nonfluorescent super‐resolution imaging.

The improved resolution obtained with the TVSFS method can be understood by analyzing the Fourier space. The bottom of Figure 1b,c depict the Fourier space for label‐free imaging and labeled imaging, respectively. The colored circles represent spectra obtained using different SFS illumination, while the circle at the center (in solid lines) represents the spectrum of an image taken with conventional microscopy. The latter spectrum contains only information from spatial frequencies less than NA/*λ*_em_ for label‐free imaging, and 2NA/*λ*_em_ for labeled imaging, where *λ*_em_ is the wavelength of the emission light. To achieve isotropic and distortionless deep super‐resolution imaging, the Fourier space needs to be enlarged and filled using omnidirectional SFS tuning, performed with enough spectral overlap. From the theoretical calculations (see Figure S3 and S4 in the Supporting Information), the spectral overlap between two successive illuminations should exceed 25%. At least three successive SFSs are required to obtain such a resolution of *λ*/4.7 for label‐free imaging and *λ*/7.1 for labeled imaging. For omnidirectional label‐free imaging, each SFS magnitude requires at least sixteen‐directional illumination, while eight‐directional illumination is needed for omnidirectional labeled imaging. On our chip, multilevel tuning is obtained using gratings with different periods and orientations. As shown in Figure 1a, well designed and period‐tunable subwavelength gratings are fabricated at predefined locations on the surface of the chip. A sample region is defined on the other side of the chip, where illumination from different directions overlap. The lateral distance between the grating and the sample region, *r*, should increase with the thickness of the chip substrate (*T*) and the angle of the light deflected by the gratings (*θ*), since *r* = *T* · tan (*θ*), as shown in the coupling map in Figure 1b,c. The gratings on the chip's surface deflect the incident propagating light, introducing evanescent waves with different wavevectors illuminated on the sample region. The SFS magnitude of an evanescent wave introduced by the gratings is defined as $k_{s}=\frac{n_{\mathrm{PC}}\cdot \sin\;\theta }{\lambda_{\mathrm{ex}}}$ for label‐free imaging and $k_{s}=\frac{2n_{\mathrm{PC}}\cdot \sin\;\theta }{\lambda_{\mathrm{ex}}}$ for labeled imaging, where *n*_PC_ is the refractive index of the photonic chip, *λ*_ex_ is the wavelength of the excitation light. Hence, small SFS magnitudes are obtained by the large‐period gratings close to the center of the chip, which can deflect the input light with a small angle. Conversely, for large SFS magnitudes, the corresponding gratings have a small period and are located near the edge of the chip. Sixteen gratings were designed for multiangle directional illumination. Hence, label‐free and labeled subwavelength resolution imaging can be realized using the same chip by switching between single‐beam illumination (Figure 1d; Figure S1, Supporting Information) and double‐beam illumination (Figure 1e; Figure S1, Supporting Information). The process for reconstructing a complete TVSFS image consists of combining spectra from different orientations from low SFS to high SFS. For label‐free TVSFS imaging, plane wave evanescent illumination with three SFS magnitudes and 16 rotational angles is made incident on the sample. The coherent interaction between this illumination and the sample generates a scattering pattern correlated to a specific part of the sample's spectrum. Label‐free TVSFS imaging of a single frame requires acquisition of 49 images (one acquisition for every SFS illumination and one for vertical illumination) of scattered patterns. The setup, physical model, and reconstruction process for label‐free TVSFS imaging are described in Figures S1 and S2 and Note S1 in the Supporting Information. With labeled TVSFS imaging, two evanescent illumination beams meet at the center of the top surface of the chip, and interfere with each other to form periodic structured light for illumination (Figure 1e). Illumination from eight angles is enabled through fabrication of an array of gratings with differing orientations, while the phases of the interference arms are controlled such that three raw images (three phases) are required for one SFS magnitude per rotational angle. The number of SFS directions required for labeled imaging is halved compared to the number needed for label‐free imaging, because the radius of the transfer function for incoherent imaging is doubled to make overlapping easier. The setup and physical model for labeled TVSFS imaging are described in Figure S1 (Supporting Information) and Note S2 in the Supporting Information, respectively.

For every frame acquired, the detected spatial spectrum can be described as

(1) $$F_{d}=F_{o}\left(\vec{k}-\vec{k_{s}}\right)\cdot \mathrm{TF}\left(\vec{k}\right)$$

where $\vec{k}=\left(k_{x},k_{y}\right)$ represents the spatial frequency in the lateral plane, $F_{o}\left(\vec{k}\right)$ is the Fourier spectrum of the object, and $\mathrm{TF}(\vec{k})$ is the transfer function of the imaging system, which is bound by the NA of the objective lens. $\vec{k_{s}}$ represents the SFS vector used in acquisition and the magnitude can be expressed as

(2) $$k_{s}=\frac{1}{p}=\frac{1}{\lambda_{\mathrm{ex}}}\;n_{\mathrm{PC}}\;\sin\;\theta =\frac{1}{\lambda_{\mathrm{ex}}}\;n_{\mathrm{PC}}\frac{r}{\sqrt{r^{2}+T^{2}}}$$

for label‐free imaging, and

(3) $$k_{s}=\frac{2}{p}=\frac{2}{\lambda_{\mathrm{ex}}}\;n_{\mathrm{PC}}\;\sin\;\theta =\frac{2}{\lambda_{\mathrm{ex}}}\;n_{\mathrm{PC}}\frac{r}{\sqrt{r^{2}+T^{2}}}$$

for labeled imaging, where *T* is the thickness of the substrate, *r* is the lateral displacement between the grating and the center of the chip, *λ*_ex_ is the wavelength of the illumination source, and *p* is the period of the gratings. Here, the refractive index of the substrate *n*_PC_ changes with the wavelength of the incident light. The illumination wavevectors can be scaled by modifying the design parameters.

Imaging resolution is determined by both the maximal aperture of the system, *k*_c_, and the SFS magnitude that is provided by the evanescent illumination module, *k*_s_, as

(4) $$\Delta_{xy}=\frac{1}{k_{c}+k_{s}}$$

where *k*_c_ is NA/*λ*_em_ and 2NA/*λ*_em_ for label‐free and labeled imaging, respectively. For label‐free imaging, the illumination wavelength equals the imaging wavelength (i.e., *λ*_em_ = *λ*_ex_).

Therefore, the theoretical resolution limit with TVSFS can be formulated as

(5) $$\Delta_{xy}=\frac{\lambda_{\mathrm{em}}}{\left(\mathrm{NA}+n_{\mathrm{PC}}\cdot \sin\;\theta \right)}$$

for label‐free imaging, and

(6) $$\Delta_{xy}=\frac{\lambda_{\mathrm{em}}}{2\left(\mathrm{NA}+\frac{n_{\mathrm{PC}}\cdot \sin\;\theta \cdot \lambda_{\mathrm{em}}}{\lambda_{\mathrm{ex}}}\right)}$$

for labeled imaging. If the SFS magnitude is larger than the *k*_c_, the resolution exceeds that of conventional wide‐field and SIM microscopy. Compared with conventional FPM, which uses real‐wavevector for multi‐SFSs, TVSFS exploits tunable virtual‐wavevector for deep‐SFSs and can achieve a resolution three times better than the Abbe diffraction limit.

The FOV of TVSFS is determined by the area of the evanescent wave, which is regulated by the area of the fabricated gratings (see Note S4 in the Supporting Information for analysis). In the present work, we obtained 40 µm × 40 µm gratings by etching with a focused ion beam (FIB), a size that can be increased to greater than 100 µm × 100 µm in future attempts. As the illumination module and the collection objective are decoupled in the TVSFS method, a low‐NA objective lens can be combined with a large‐wavevector illumination module to simultaneously achieve a high resolution and a large FOV.

**Figure 2:**
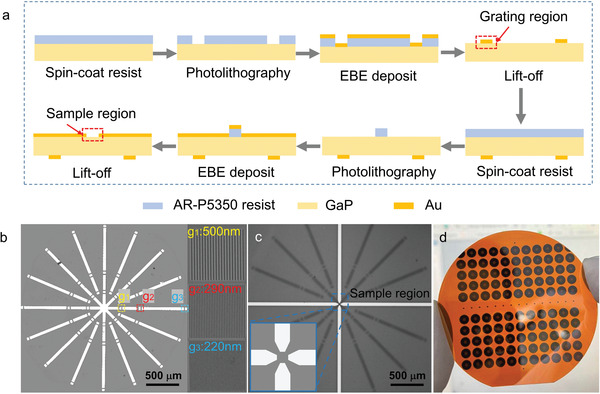
Wafer‐scale fabrication of a TVSFS super‐resolution imaging chip. a) Schematic explanation of the wafer‐scale chip‐fabrication process. b) Optical microscopy image of the bottom surface of the chip. Insets: Gratings with periods of 500 nm (g_1_), 290 nm (g_2_), and 220 nm (g_3_), corresponding to the labels in the main figure. c) Optical microscopy image of the top surface of the chip. Inset: Enlarged view of the sample region. d) Array of super‐resolution imaging chips fabricated on a 2 in. GaP wafer.

In this work, we created arrays of TVSFS chips on a two‐inch wafer using standard photolithography and lift‐off processes. Background light suppression is the crucial step in successful TVSFS imaging, as the coupling of light with the gratings introduces stray reflections. To achieve this suppression, we developed a light‐blocking chip, fabricated using dual surface lithography followed by metal deposition, as summarized in **Figure** **2**a. The bottom surface of the chip is for spatial frequency shifting, achieved with the multilevel gratings, while the top surface is for imaging. A modified chip design with more light blocking is demonstrated in Figure S10 (Supporting Information). All areas on the chip surfaces other than the sample or gratings regions are covered with metal to block the background light. Besides blocking the stray light, the metal film also absorbs the evanescent wave that incidents onto it. Therefore, the light bounces back at boundaries outside the field of view can be neglected, and we can get raw images with high contrasts. In addition to providing an unlimited FOV, the transparency of the GaP substrate for visible wavelengths makes a dual surface lithography process using a standard lithography machine possible. We used FIB to fabricate the different gratings at the predesigned positions. Optical images of the top and bottom surfaces of a chip following fabrication are shown in Figures 2b,c. With this process, more than 100 chips can be fabricated in a batch and separated for use.

**Figure 3:**
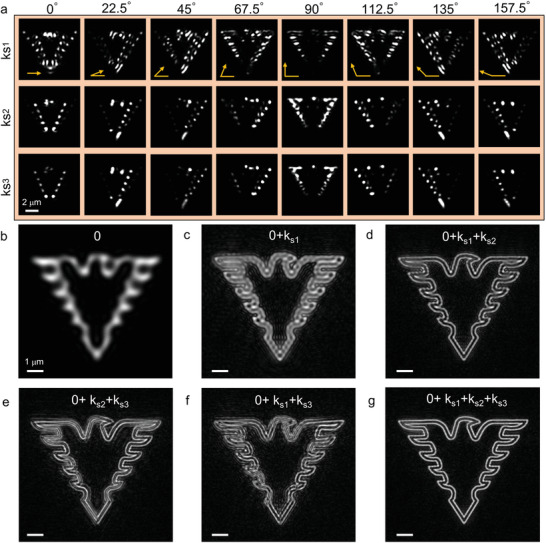
Simulation of label‐free TVSFS imaging. a) Spatial domain representations of different angles of the Fourier spectrum of a pattern of Zhejiang University's eagle logo. b) Conventionally acquired wide‐field image containing only low‐frequency information. c–g) TVSFS reconstruction with different parts of the Fourier spectrum. Scale bars: (a) 2  µm; (b–g) 1 µm.

To investigate the label‐free imaging capability of our TVSFS chip, we simulated multilevel tuning of the illumination wavevector. Here, a pattern of Zhejiang University's eagle logo, defined with a double‐line profile with line‐to‐line distance set as 215 nm, was selected as the imaging sample. The spatial distribution of this pattern under multilevel wavevector illumination provided by a 660 nm source is presented in **Figure** **3**a. Here, *k*_s1_ − *k*_s3_ represent SFS vectors with increasing magnitudes (1.3*k*_0_, 2.3*k*_0_, and 3.1*k*_0_), and $k_{0}=\frac{1}{\lambda_{\mathrm{ex}}}$ is the magnitude of SFS vector in free space. These SFS vectors were selected such that there was enough overlap between them in the Fourier space to ensure good convergence during image reconstruction. A conventionally acquired image of the eagle logo, obtained with vertical illumination through a 0.85‐NA objective, is shown in Figure 3b, while the image in Figure 3g depicts a reconstruction covering the low‐SFS, middle‐SFS, and high‐SFS spectrum band. Only the latter image reproduces the double‐line details, highlighting the improved resolution obtained with multilevel tuning. In contrast, omitting parts of the spectrum, i.e., without multilevel tuning, results in images with poorer resolution (Figure 3c,d), or false reconstruction of the actual sample (Figure 3e,f).

**Figure 4:**
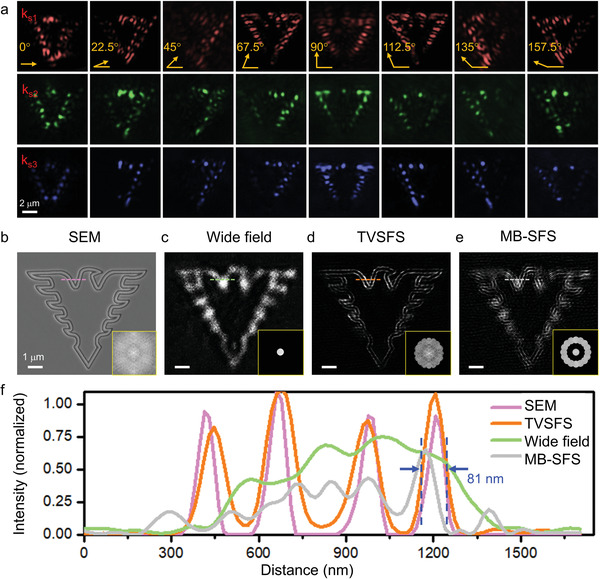
On‐chip label‐free TVSFS imaging of etched “ZJU” eagle logo. a) Raw label‐free TVSFS images acquired from eight different directions using three wavevectors of varying magnitude. Colors indicate the magnitude of the wavevector used for image acquisition (*k*_s1_, *k*_s2_, and *k*_s3_ indicate SFS magnitudes of 1.3*k*_0_, 2.3*k*_0_, 3.1*k*_0_, respectively). Images of the eagle logo taken using b) SEM, c) conventional wide‐field microscopy under vertical illumination, d) label‐free TVSFS imaging, and e) label‐free MB‐SFS. Insets in the bottom‐right corner of these figures correspond to the Fourier spectra of the acquired images. f) Line profiles of the region indicated by the dashed line in (b)–(e). The intensity profiles of the SEM and wide‐field images were inverted for better correspondence to the dark‐field TVSFS image. Details of the etched lines that are not resolved in the wide‐field image (green line) and MB‐SFS image (gray line) are clearly resolved in the TVSFS image (red line). The line profile of the latter image matches well with that of the SEM image (magenta line). Scale bars: (a) 2  µm; (b–e) 1 µm.

To confirm this behavior, we conducted practical experiments recreating the simulations, using the same parameters for comparison. The eagle logo was etched on the surface of a GaP wafer using FIB, as shown in the scanning electron microscopy (SEM) image in **Figure** **4**b. We used a 660 nm wavelength laser diode for illumination. Diode excitation was modulated using a sawtooth wave, and the resulting laser beam was spatially filtered, to suppress speckle noise and improve the signal‐to‐noise ratio (SNR) of the raw images (see Figure S5 in the Supporting Information). Experimentally acquired images (Figure 4a) matched well with those obtained in simulations (Figure 3a). The minor mismatches between experimental and simulated images can be explained by fabrication defects, since the uniformity of grating fabrication and the refractive index of the photonic substrate are crucial for obtaining a perfect raw image. The final high‐resolution image of the sample (Figure 4d) was obtained using our spatial spectrum splicing algorithm (see Note S1 in the Supporting Information). A 4.5× resolution enhancement relative to the resolution of images acquired directly with wide‐field illumination can be observed with the label‐free TVSFS images (see the Fourier spectra at the bottom‐right corner of Figure 4b–d). There is an excellent match between the line profiles of the SEM and TVSFS images (Figure 4f), validating our method. The image of missing spectrum band SFS (MB‐SFS) presents false reconstruction and inferior match with the SEM image (Figure 4e,f). Image resolution can be increased further using a shorter wavelength. This is demonstrated in Figure S6 (Supporting Information), which depicts a sample etched with four lines imaged with 561 nm illumination, such that resolution of lengths as small as 120 nm was achieved.

Unlike label‐free imaging where uniform evanescent waves provide coherent illumination, labeled fluorescent imaging requires periodic patterns formed by the interference between two counterpropagating evanescent waves.^[^ ^28^ ^]^ The counterpropagating evanescent waves used in our experiments were obtained by stimulating grating couples. Both beams were TE‐polarized, corresponding to the orientation of the gratings (see Figures S7 and S8 in the Supporting Information), to enhance the contrast of the interference pattern, which determines the resolution of the final reconstructed image. SFS magnitude is adjusted by changing the grating period. The photonic chips were fabricated with an empty sample region and three periods of gratings (see Figure S10 in the Supporting Information). Each period has eight gratings with equally distributed azimuthal angles. The gratings were distributed at predefined positions to ensure that light with SFS magnitudes of 1.8*k*_0_, 3.2*k*_0_, and 4.8*k*_0_ overlapped in the sample region. The SFS magnitudes were designed to ensure enough spectrum overlap between the adjacent shift values (see Figure S4 in the Supporting Information). A 1.1‐NA water‐immersion objective lens was used to collect the raw TVSFS images. Light splitting and phase shifting (three phases per orientation per SFS magnitude) between the two light paths were achieved by controlling the laser beam using a spatial light modulator (SLM). For isotropic resolution, the SLM pattern has to be rotated to have four equally spaced orientations. For higher resolutions, the distance between the two beams should be adjusted to fit the gratings with smaller periods (distributed at the margin of the photonic chip).

**Figure 5:**
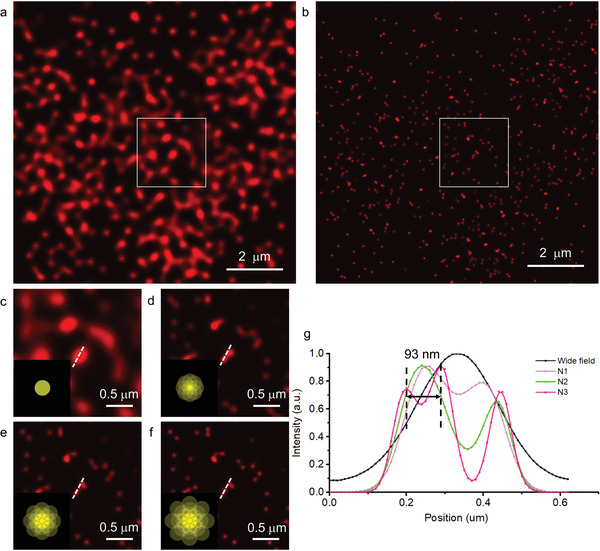
Resolving fluorescent beads with labeled TVSFS imaging. a) Diffraction‐limited image of a sample of fluorescent 40 nm beads. b) TVSFS reconstruction of (a). c–f) Enlarged views of the area enclosed by the white box in (b), where (c) is the wide‐field image, and (d–f) show the images reconstructed using the TVSFS method with maximum SFS magnitude of 1.8*k*_0_, 3.2*k*_0_, and 4.8*k*_0_. Insets in the bottom left corners of these figures show the corresponding Fourier spectra. g) Line profiles of the region indicated by the white line in (c)–(f). Two beads located 93 nm apart are resolved individually in the labeled TVSFS image with a maximum SFS magnitude of 4.8*k*_0_, but not in the diffraction‐limited image. All images were obtained with excitation/emission wavelengths of 639 nm/661 nm.

To demonstrate labeled TVSFS imaging, we conducted experiments using fluorescent 40 nm beads. Samples were illuminated by periodic patterns with varying fringe spacings, and reconstructed sequentially to incorporate higher SFS magnitudes (the complete reconstruction method is detailed in Note S2 in the Supporting Information). Conventionally acquired images of the fluorescent beads are shown in **Figure** **5**a,c, while Figure 5b,d–f depict reconstructed images. The line profiles (Figure 5g) from the regions marked with the white dashed lines in Figure 5c–f illustrate how resolution improves as larger wavevectors are considered. Finer details can be observed clearly in the TVSFS reconstruction in a region that appears as a single feature in the diffraction‐limited image (Figure 5c). The intensity profiles indicate that image contrast is improved when the SFS magnitude increases from 1.8*k*_0_ to 3.2*k*_0_, and individual beads can be resolved when the SFS magnitude reaches 4.8*k*_0_. Based on the line profile in Figure 5g, the two beads in Figure 5f are separated by 93 nm.

**Figure 6:**
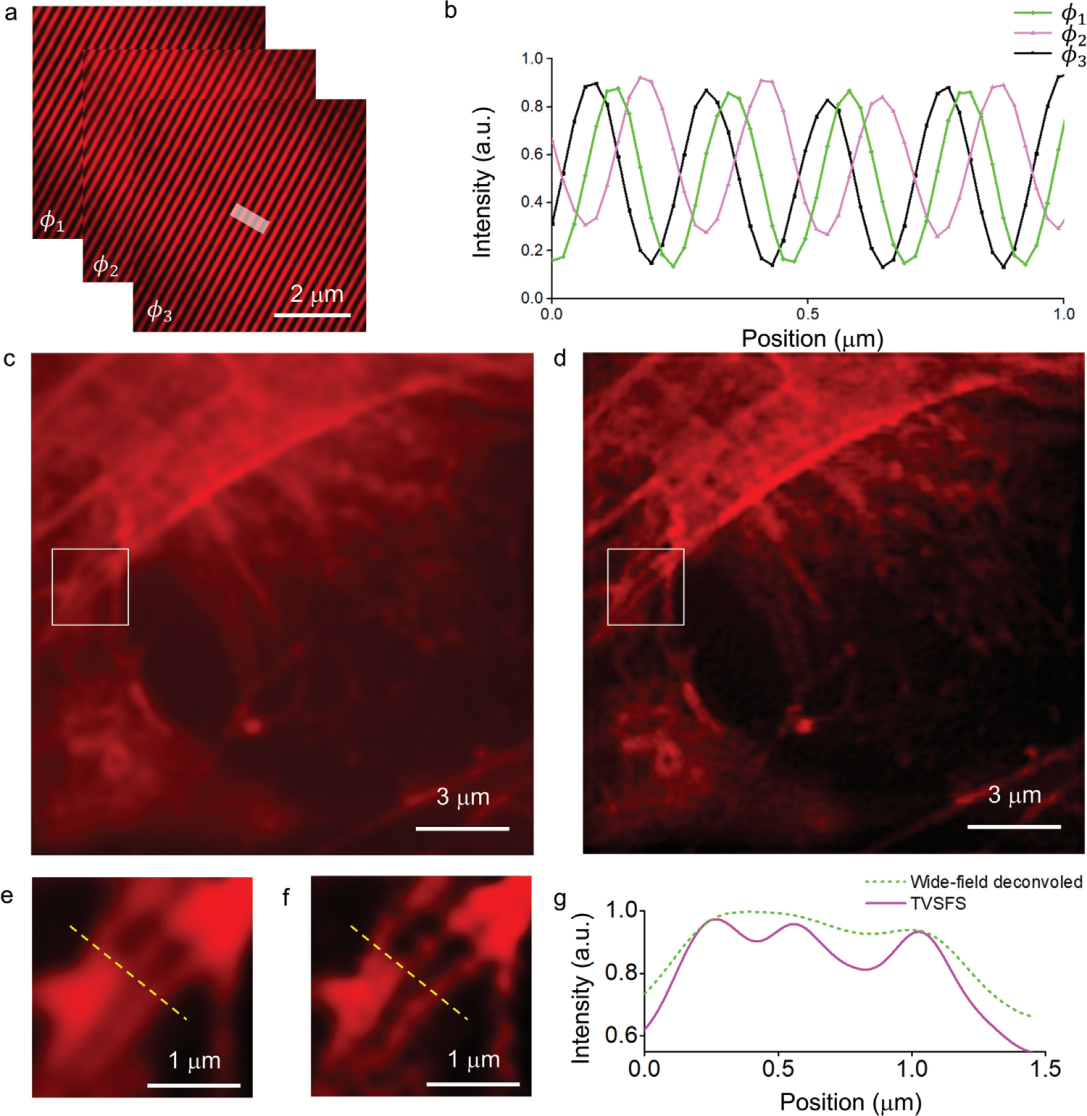
Experimental demonstration of labeled TVSFS imaging of U2OS. a) Illumination patterns for labeled TVSFS imaging generated by the photonic chip. The counterpropagating light was designed to have a SFS magnitude of 2.96*k*_0_. The polarization direction was changed to maximize the pattern contrast. The resulting interference patterns were imaged using a 1.49‐NA objective. b) Intensity profile along the line in a. The period of these patterns is 215 nm, indicating an evanescent illumination wave with a refractive index corresponding to 1.48. c) Wide‐field fluorescent image of a U2OS cell (human osteosarcoma cell line). d) Reconstructed TVSFS image of the same cell, demonstrating clear resolution enhancement compared to (c). e) Enlarged image of the area enclosed by the white box in (c). f) Enlarged image of the area enclosed by the white box in (d). g) Line profile of the location marked by the yellow dashed lines in (e) and (f).

Finally, we tested the feasibility of imaging biological specimens (human bone osteosarcoma epithelial cells (U2OS)) using the on‐chip TVSFS platform. As the cells selected adhere to GaP surfaces excellently, the sample preparation processes for TVSFS imaging are identical to those used with a conventional glass slide; cells were deposited on the surface of the TVSFS chip, following which, their actin filaments were labeled using Alexa Fluor 647 phalloidin (a detailed summary of this procedure is included in the materials and methods section). Imaging was conducted using a 1.49‐NA objective lens in combination with a chip with a SFS magnitude of 2.96*k*_0_. The aberration of the illumination patterns plays a vital role in the final reconstruction. It has been suggested that the presence of biological samples within the region of evanescent penetration can cause such aberrations. We overturned this conjecture by capturing the light propagated through the biological samples (**Figure** **6**a). The image depicts a near‐perfect low‐intensity recreation of three phase‐shifted illumination patterns propagating in one direction. Based on a line profile of the region indicated by the white box in Figure 6a, the phase shift of these patterns is ±120° (Figure 6b), which is accurate to the designed shift. For high‐resolution image reconstruction, we acquired spatial‐frequency‐shifted wide‐field images with three phase shifts in three directions, by adjusting the patterns on the SLM nine times. The actin filaments, which are not discernable in the wide‐field image (Figure 6c,e) can be seen in greater detail in the reconstructed TVSFS images (Figure 6d,f). This result thus demonstrates that the chip‐based TVSFS method can be used for imaging biological samples without any modification to existing sample preparation protocols.

## 3 Discussion and Conclusion

In this work, we demonstrated a universal super‐resolution imaging method based on the TVSFS effect. The TVSFS method can be implemented using a high‐refractive‐index photonic chip, to provide large‐FOV, deep super‐resolution imaging. Theoretical modeling of the TVSFS principle, complemented and confirmed by experimental work, demonstrates the potential of the proposed TVSFS chip for use with both labeled and label‐free samples. Three elements are key to this achievement: 1) a photonic chip with a high refractive index, to provide a virtual‐wavevector SFS; 2) micro/nanostructures of well‐designed and tuned sizes fabricated on the surface of the chip, for tunable multilevel spatial frequency shifts; and 3) compatibility with single‐beam and double‐beam illumination, for switching between labeled and label‐free imaging. In addition, for a large FOV, the different beams must overlap accurately. This accuracy can be achieved by controlling the size and position of the micro/nanostructures precisely during fabrication.

Our TVSFS technique has many advantages over existing on‐chip super‐resolution methods. As well as precise and flexible control of the SFS vector, it is compatible with label‐free and labeled imaging, unlike techniques such as nanowire ring illumination microscopy,^[^ ^12^, ^29^ ^]^ and fluorescent polymer film‐based SFS chips,^[^ ^13^ ^]^ which are exclusively label‐free. Although the SFS magnitude can be tuned when integrated optical waveguide‐based methods such as cSIM^[^ ^14^ ^]^ are used, since SFS tuning is performed by adjusting the azimuthal interference angle, unlike our technique, they are only compatible with labeled imaging, because the magnitude of the illumination wavevector cannot be adjusted freely. Using other multiwavelength schemes^[^ ^15^, ^16^, ^17^ ^]^ in combination with high‐refractive‐index materials, such as GaP and plasmonic materials, results in the loss of some spectrum bands. While deep‐subwavelength resolution can be achieved with waveguide‐based fluorescent methods such as waveguide‐based points accumulation in nanoscale topography (PAINT)^[^ ^26^ ^]^ and chip‐based (direct) stochastic optical reconstruction microscopy ((d)STORM),^[^ ^25^ ^]^ the TVSFS method offers the possibility of more efficient high‐speed imaging; as reconstruction is performed in the Fourier space, a large FOV can be recovered with a smaller number of frames (see Note S6 in the Supporting Information for analysis). Furthermore, the TVSFS method is compatible with low illumination intensities and standard fluorescence imaging.

The TVSFS photonic chip offers universal super‐resolution imaging with user‐defined illumination wavevectors. The parameters of our chip can be retrofitted easily, allowing super‐resolution imaging with standard microscopy equipment, while large arrays of chips can be fabricated on a wafer, which is appropriate for increasing the scale and reducing the cost of production. The single TVSFS photonic chip can be integrated with a small optical collection system (such as a smartphone with a camera), to function as a light‐weighted super‐resolution imaging system.

The resolution of the TVSFS method is closely related with two parameters: the maximum lateral wavevector magnitude of the illumination and the SNR of collected raw images. As shown in Table S1, SiC is almost transparent at the shorter wavelength with a high refractive index. Therefore, using SiC as the substrate combined with a shorter wavelength can provide a higher resolution. Besides natural materials, a much larger lateral wavevector could be achieved by using hyperbolic metamaterials,^[^ ^30^ ^]^ which support a much higher resolution down to less than 10 nm. Similar to other super‐resolution methods like STORM, the ultimate resolution of the TVSFS method is only limited to the SNR of the images we can acquire.

Practically, the high‐SFS signal for normal samples is much weaker than that of low‐SFS, and the quality of collected raw images will be affected by the photon noise. In the simulation, the decrease of SNR will degrade the resolution achieved, as demonstrated in Figures S12 and S13 (Supporting Information). A lower photon number will cause an unsmooth connection in the Fourier space, and for both labeled and label‐free imaging, only when the maximum photon number reaches 1000 or more, the theoretical resolution can be achieved. The input power of the illumination could be increased to achieve a better SNR, which may bring the problem of camera saturation for the low‐SFS raw images. Therefore, for low‐SFS and high‐SFS, the illumination intensity should be adjusted and the spectrum signal be rescaled accordingly in the final spectrum splicing process. Another concern regarding the method is that, although resolution can be improved by increasing the lateral wavevector magnitude of the illumination, the resultant shallower field penetration depth would limit the capability for volumetric imaging. Thus, the resolution and imaging depth need to be optimized together, according to the specific application. From another perspective, the shallow illumination depth also brings the advantage of reduced defocus noise, making the TVSFS method suitable for imaging structures located near the chip surface, for example, the cell membrane.

Our TVSFS super‐resolution technique can be improved further in a number of ways. First, the number of SFS magnitudes required for good image reconstruction can be reduced with the adoption of better reconstruction methods, such as deep‐learning techniques,^[^ ^31^, ^32^ ^]^ thus decreasing the numbers of gratings to be fabricated and raw images to be captured (see Note S6 in the Supporting Information for analysis). This consequently reduces the cost of chip fabrication and increases the speed of imaging. Second, the errors in the necessarily imperfect geometries of the gratings may cause two effects: the nonuniformity of the illumination intensity and the introduction of impure wavevectors, which will reduce the resolution and introduce artifacts using the conventional reconstruction algorithm. However, these effects on the image can be relieved by using pattern‐estimation reconstruction methods, such as blind SIM.^[^ ^33^ ^]^ Thirdly, advanced fabrication techniques may also improve the performance of the TVSFS chip. For instance, the FOV can be enlarged by fabricating large arrays of micro/nanostructures using a technique such as nanoimprint lithography. With minor design changes, an on‐chip evanescent light source can be implemented on the device to replace the off‐chip light source. Commercial LEDs or vertical‐cavity surface‐emitting laser (VCSEL) chips^[^ ^34^ ^]^ can be bonded to the photonic chip, thereby providing cheap, mass‐producible, microscope‐compatible, multimode super‐resolution imaging. In the future, the highly integrated TVSFS chip may serve as a multifunctional platform on which many functions (e.g., electrical stimulation, microfluidics, and sensing) can be integrated for application in fields such as biology, materials science, and chemical research.

## 4 Experimental Section

### Chip Preparation

A double‐polished 2 in. diameter GaP wafer with a thickness of 500 µm was bought from the Hefei Kejing Materials Technology Company. The position of the gratings was designed using Matlab (see Note S3 in the Supporting Information for the error analysis) and defined using standard photolithography and lift‐off processes. After spin‐coating the surface of the GaP wafer with AR‐P5350 resist, photolithography was performed using the MA6‐BSA mask aligner from Karl Suss. A Cr film was subsequently deposited on the GaP substrate, followed by a 50 nm thick gold film, with the final patterns revealed following lift‐off. Gratings were fabricated in this pattern using FIB (Quanta 3D FEG, Carl Zeiss) with a beam current of 20 pA. An additional photolithography and lift‐off process were conducted on the other side of the substrate, marking the sample region with a Cr pattern, to block stray light. The Cr films were deposited by magnetron sputtering using the DISCOVERY‐635 from DENTON, while the gold film was deposited using electron beam evaporation.

### Experimental Setup

The imaging setup (see Figure S1 in the Supporting Information) was based on a custom‐built upright microscope (Cerna, Thorlabs) fitted with an sCMOS camera (ORCA flash or Prime95B, Hamamatsu). This was equipped with a piezo objective scanner (ZFM2020, Thorlabs) for adjusting the focal plane of the objective lens, and a three‐degrees‐of‐freedom linear translation stage (M‐462, Newport) for coarse alignment of the chip with respect to the objective lens. The laser beams were expanded by a 4‐f system (L1 and L2) to fill the LCOS‐SLM (X13139‐01, Hamamatsu), and relayed to the microscope by another 4‐f lens (L3 and L4). L5 was used to focus the light onto the gratings of the photonics chip. TVSFS images were acquired using a Zeiss ×100/0.85 air, an Olympus ×100/1.1 water, or an Olympus ×100/1.49 oil objective lens. For label‐free imaging, excitation from the 660 nm laser diode (L650P007, Thorlabs) was modulated by a sawtooth wave from a function generator (Stanford DS345), or by focusing the 561 nm laser beam (MGL‐FN‐561, Changchun New Industries Optoelectronics Technology Company) through rotating ground glass. A 300 mW 639 nm laser (MSL‐FN‐639, Changchun New Industries Optoelectronics Technology Company) was used as the excitation source for labeled imaging, with a 661 nm bandpass filter (87‐755, Edmund Optics) blocking emission from unwanted wavelengths. A half‐wavelength plate (HWP) was inserted between the laser and the SLM to adjust the polarization of the light beam, while the combination of the quarter wavelength plate (QWP) and the liquid crystal retarder were used to optimize the light polarization incident on the gratings.

### Sample Preparation

The GaP chip was washed several times with ethyl alcohol and ultrapure water, and sterilized for 30 min in ultraviolet light. U2OS (human osteosarcoma cell line; ATCC) cells were cultured in McCoy's 5A medium (Thermo Fisher Scientific Inc.) supplemented with 10% (v/v) fetal bovine serum (Thermo Fisher Scientific Inc.). Before labeling, the cells were seeded on the GaP chip and incubated overnight at 37 °C in a humidified 5% CO_2_ environment. After incubation, the cells were washed three times with phosphate buffered saline (PBS; Thermo Fisher Scientific Inc.), fixed with 4% paraformaldehyde (Electron Microscopy Sciences) for 10 min, and incubated with Alexa Fluor 647 phalloidin (Thermo Fisher Scientific Inc.) for 30 min at 37 °C, according to the manufacturer's instructions. The cells were then washed three times with PBS and mounted in ProLong Diamond Antifade Mountant (Thermo Fisher Scientific Inc.).

For nonbiological imaging, a 1 µL drop of fluorescent 40 nm diameter beads (FluoSpheres F8789, Thermo Fisher Scientific Inc.) with a concentration of 0.005% was placed on the sample region using a pipette, and dried with nitrogen. The chip was then put in a cell culture dish for water immersion imaging.

### Simulation

Simulations were implemented using home‐built algorithms in Matlab software. The algorithms for modeling TVSFS label‐free and labeled imaging are discussed in detail in Note S1 and S2 in the Supporting Information, respectively.

## Conflict of Interest

The authors declare no conflict of interest.

## Supporting information

Supporting Information

## Data Availability

The data that support the findings of this study are available from the corresponding author upon reasonable request.
