# Supplementary material for: High‐Refractive‐Index Chip with Periodically Fine‐Tuning Gratings for Tunable Virtual‐Wavevector Spatial Frequency Shift Universal Super‐Resolution Imaging
Source: Adv Sci (Weinh). 2022 Jan 27;9(9):2103835. doi: 10.1002/advs.202103835 (PMC8948578; doi:10.1002/advs.202103835)
Supplement: Supplementary file 1 — Supporting Information [file ADVS-9-2103835-s001.pdf]

ADVANCED SCIENCE
Open Access

Supporting Information

for *Adv. Sci.*, DOI 10.1002/advs.202103835

High-Refractive-Index Chip with Periodically Fine-Tuning Gratings for Tunable Virtual-Wavevector Spatial Frequency Shift Universal Super-Resolution Imaging

Mingwei Tang, Yubing Han, Dehao Ye, Qianwei Zhang, Chenlei Pang, Xiaowei Liu, Weidong Shen, Yaoguang Ma, Clemens F. Kaminski, Xu Liu and Qing Yang**

ADVANCED SCIENCE
Open Access

Supporting Information

for *Adv. Sci.*, DOI: 10.1002/advs.202103835

High-refractive-index chip with periodically fine-tuning gratings for tunable virtual-wavevector spatial frequency shift universal super-resolution imaging

Mingwei Tang, Yubing Han, Dehao Ye, Qianwei Zhang, Chenlei Pang, Xiaowei Liu, Weidong Shen, Yaoguang Ma, Clemens F. Kaminski, Xu Liu and Qing Yang**

Supporting Information

High-refractive-index chip with periodically fine-tuning gratings for tunable virtual-wavevector spatial frequency shift universal super-resolution imaging

Mingwei Tang,[1] Yubing Han,[1] Dehao Ye,[1] Qianwei Zhang,[1] Chenlei Pang,[1,2] Xiaowei Liu,[1,2] Weidong Shen,[1] Yaoguang Ma,[1] Clemens F. Kaminski,[3] Xu Liu,[1,2,4,]* and Qing Yang[1,2,4,]*

[1] *State Key Laboratory of Modern Optical Instrumentation, College of Optical Science and Engineering, International Research Center for Advanced Photonics, Zhejiang University, Hangzhou 310027, China*

[2] *Research Center for Humanoid Sensing, Zhejiang Lab, Hangzhou 311100, China.*

[3] *Department of Chemical Engineering and Biotechnology, University of Cambridge, Cambridge, CB30AS, United Kingdom*

[4] *Collaborative Innovation Center of Extreme Optics, Shanxi University, Taiyuan, 030006, China*

**Corresponding authors: liuxu@zju.edu.cn, qingyang@zju.edu.cn*

Table of content

Supplementary Note 1: Physical models and reconstruction process of TVSFS label-free SR imaging. 3
Supplementary Note 2: Physical models and reconstruction process of TVSFS labeled imaging. 6
Supplementary Note 3: Error analysis of the chip design....10
Supplementary Note 4: Determining the FOV of TVSFS method. 12
Supplementary Note 5: Performance of TVSFS imaging with photon noise. 13
Supplementary Note 6: Imaging speed of TVSFS method. 14
Figure S1. Schematic of chip-based TVSFS imaging 16
Figure S2. Iterative recovery procedure of TVSFS label-free imaging. 17
Figure S3. Reconstructions of TVSFS label-free imaging with different spectrum overlapping percentages. 18
Figure S4. Reconstructions of TVSFS labeled imaging with different spectrum overlapping percentages. 19
Figure S5. Comparison of TVSFS label-free raw images with or without laser modulation. 20
Figure S6. Resolution calibration of TVSFS label-free imaging using the 561 nm laser and 1.49-NA objective. 21
Figure S7. Polarization control in TVSFS label imaging 22
Figure S8. Polarization control in TVSFS labeled imaging 23
Figure S9. Error analysis of the chip fabrication 24
Figure S10. Modified chip design for TVSFS imaging with more light blocking 25
Figure S11. FOV determination for TVSFS imaging 26
Figure S12. Performance of label-free imaging with Poisson noise. 27
Figure S13. Performance of labeled imaging with Poisson noise. 28
Table S1. The refractive index, extinction coefficient of GaP[7] and 4H-SiC,[8] and the ultimate resolution for various wavelengths with NA of 1.49. 29

Supplementary Note 1: Physical models and reconstruction process of TVSFS label-free SR imaging.

As shown in **Figure S1** , in our TVSFS label-free method, the sample holder and evanescent wave generator are integrated into a single chip. The TVSFS label-free imaging method follows the coherent imaging model, which assumes the filtering function of the objective lens (that is the CTF) is a circular pupil with a radius of NA/λ_{em}.

Suppose a thin sample on the chip surface has a spatial distribution of $\psi(r)$. On the other side of the chip, gratings functioning as the plane wave source have the illumination position r_i. For a photonics chip with a refractive index of n_{PC}, the sample's complex field is:

$$S(r) = \psi(r)e^{jk\cdot n_{PC}\cdot sin\theta\cdot r} \qquad S\,1$$

where the effective refractive index $n_{PC}sin\theta$ is related to the light propagation angle θ in the photonic chip. $sin\theta$ is decided by the wafer thickness T and shift illumination position r_i:

$$sin\theta = \frac{r_i}{\sqrt{r_i^2 + T^2}} \qquad S\,2$$

$S(r)$ continues to propagate through the objective lens with an aperture transmittance function of CTF(k). The process can be mathematically expressed by the Fourier transform of $S(r)$ and subsequently multiplied by CTF(k), creating the optical field:

$$\mathcal{F}[S(r)]CTF(k) = \tilde{\psi}(k - n_{PC}\cdot sin\theta)\cdot CTF(k) \qquad S\,3$$

Finally, the light intensity imaged on the camera can be expressed as:

$$I_{k_s,\phi}(k) = \left|\mathcal{F}^{-1}\left[\tilde{\psi}(k - n_{PC}\cdot sin\theta)\cdot CTF(k)\right]\right|^2 \qquad S\,4$$

Let the raw images be denoted as $I_{k_s,\phi}(k)$, with k_s the SFS magnitude and ϕ the azimuthal angle, respectively. As we can see from the equation, the detected intensity information contains the spatial spectrum of the sample shifted by a wavevector determined by the effective index of the illuminated wave $n_{PC}\cdot sin\theta$, and then low-pass filtered by the CTF of the objective lens. The high refractive index of the wafer makes $|n_{PC}\cdot sin\theta|$ exceed that can be obtained in the air, which will bring much deeper spatial spectrum of the object to be collected by the optical system.

As indicated in Equation 4, the best resolution is determined by k_c and k_s, where k_c is maximal aperture of the system and k_s is the SFS magnitude that is provided by the evanescent

illumination module.

For label-free imaging, we use the coherent imaging model, so that

$$k_c = \frac{NA}{\lambda_{em}} \quad S\,5$$

$$k_s = \frac{n_{PC} \cdot sin\theta}{\lambda_{ex}} \quad S\,6$$

For label-free TVSFS imaging, $\lambda_{em} = \lambda_{ex}$. By adopting Equation 4, we can deduce

$$\Delta_{xy} = \frac{\lambda_{em}}{(NA + n_{PC} \cdot sin\theta)} \quad S\,7$$

The principal of the TVSFS label-free recovery process is retrieving the phase map from multiple intensity images,[1] which alternates between the spatial domain (*x*-*y*) and the spatial frequency domain ($k_x - k_y$), as shown in Figure S2.

(Step 1): The recovery process begins with making a guess of the super-resolution image in the spatial domain: $\sqrt{I_h}e^{i\varphi_h}$.The initial guess will not fail the reconstruction but a good guess will affect the convergence rate and the signal-to-noise rate (SNR) of the final image. Usually the wide field low-resolution image is used to get a fast convergence but this will bring many noises in the background of the Fourier space. Here we use the all-one image as the guess to get a clean spatial frequency background.

(Step 2) Second, we use the CTF to select the specific spatial spectrum and apply inverse Fourier transformation to generate a new low-resolution target image $\sqrt{I_l}e^{i\varphi_l}$. This process also keep noise from outside the pass-band of the objective.

(Step 3) The phase φ_l of the low-resolution image is used as the estimate of the phase map φ_h of the sample $S(x)$. Therefore, only the amplitude of the low-resolution image is updated by the intensity measurement $I_{k_s,\phi}$, that is $\sqrt{I_l}e^{i\varphi_l} \rightarrow \sqrt{I_{k_s,\phi}}e^{i\varphi_l}$. The complex image is then applied with Fourier transformation, and used to replace the subregion in the spatial spectrum. This step can be expressed as:

$$F^{m+1}(k) = F^m(k) \times \left(1 - CTF(k - k_s)\right) + CTF(k - k_s) \times \mathcal{F}\left(\sqrt{I_{k_s,\phi}}e^{i\varphi_l}(k - k_s)\right) \quad S\,8$$

(Step 4) In the next step, we repeat step 2 and 3 to update the spatial spectrum with all the raw

images. The images number is the multiplication of the number of SFS magnitudes $N(k_S)$ and the number of azimuthal angles $N(\phi)$.

(Step 5) Finally, steps 2-4 were repeated several times until the convergence condition is satisfied. To get the intensity of the sample, we inverse Fourier transform the spatial spectrum and get the sample's complex field $\sqrt{I_h}e^{i\varphi_h}$. The intensity distribution of the thin sample is the amplitude $\sqrt{I_h}$.

The presented algorithm was tested on simulated data, as displayed in the top of Figure S2 for a ground truth input and a successful intensity reconstruction.

Supplementary Note 2: Physical models and reconstruction process of TVSFS labeled imaging.

1. Physical models of TVSFS labeled imaging

The TVSFS labeled imaging uses the periodic patterns formed by interference between lights coupled from two opposite input gratings to illuminate the fluorescently labeled samples. This imaging process can be expressed as:

$$I_{ex}(\vec{r}) = A_1(\vec{r})^2 + A_2(\vec{r})^2 + 2A_1(\vec{r})A_2(\vec{r})\, cos\left[\left(\overrightarrow{K_1} - \overrightarrow{K_2}\right)\cdot \vec{r} + \Delta\phi\right] \qquad S\,9$$

where $A_1(\vec{r})$ and $A_2(\vec{r})$ represent the amplitudes of the two input lights; $\overrightarrow{K_1}$ and $\overrightarrow{K_2}$ represent their propagation wave vectors; $\Delta\phi$ represents their phase difference. $\left|\overrightarrow{K_1}\right| = \left|\overrightarrow{K_2}\right| = 2\pi\frac{n_{eff}}{\lambda_{ex}}$. The phase difference $\Delta\phi$ should be changed three times per azimuthal angle per SFS magnitude. The first and second items in Equation *S 9* represents the background, and the third item represents a structured illumination with spatial-frequency $\Delta\vec{K} = \vec{K}_1 - \vec{K}_2$. The background can be calibrated

$$I = \frac{I_{raw}}{[A_1(\vec{r})^2 + A_2(\vec{r})^2]} \qquad S\,10$$

using Equation *S 10*:

$$I = \left\{1 + \frac{2A_1(\vec{r})A_2(\vec{r})}{[A_1(\vec{r})^2 + A_2(\vec{r})^2]} cos\left[(\Delta\vec{K})\cdot\vec{r} + \Delta\varphi\right]\right\}\cdot O * PSF \qquad S\,11$$

The calibrated image intensity distribution I can be expressed as:

where $*$ represents convolution operation, O represents object distribution, and PSF represents the point spread function of the detection system, which is the Fourier transform of the optical transfer function (OTF). To analyze the Fourier components of the image, we make a Fourier transform to Equation *S 11*.

$$\begin{aligned}\tilde{I} &= \left\{\tilde{O} + \mathcal{F}\left\{\frac{2A_1(\vec{r})A_2(\vec{r})}{[A_1(\vec{r})^2 + A_2(\vec{r})^2]}\right\} * \mathcal{F}\left[cos\left(\Delta\vec{K}\cdot\vec{r} + \Delta\varphi\right)\right] * \tilde{O}\right\}\cdot OTF \\ &= \left\{\tilde{O} + \tilde{M} * \left[0.5e^{i\Delta\varphi}\delta\left(\vec{K} - \Delta\vec{K}\right) + 0.5e^{-i\Delta\varphi}\delta\left(\vec{K} + \Delta\vec{K}\right)\right] * \tilde{O}\right\}\cdot OTF \qquad S\,12 \\ &= \left\{\tilde{O} + 0.5e^{i\Delta\varphi}\tilde{M}(\vec{K} - \Delta\vec{K}) * \tilde{O} + 0.5e^{-i\Delta\varphi}\tilde{M}(\vec{K} + \Delta\vec{K}) * \tilde{O}\right\}\cdot OTF\end{aligned}$$

$$= \tilde{O}^0 + 0.5e^{i\Delta\varphi}\tilde{G}^+ + 0.5e^{-i\Delta\varphi}\tilde{G}^-$$

where $\mathcal{F}$ represents Fourier transform operator; $\tilde{M} = \mathcal{F}\left\{\frac{2A_1(\vec{r})A_2(\vec{r})}{[A_1(\vec{r})^2+A_2(\vec{r})^2]}\right\}$; $\tilde{G} = \tilde{M} * \tilde{O}$. The superscripts "0,+,−" represents the components centered at origin (zero-order component), $\Delta\vec{K}$ and$-\Delta\vec{K}$ (shifted components) respectively. $\tilde{O}^0 = \tilde{O} \cdot OTF$ is the spatial-frequency component of conventional wide-field image. $\tilde{G}^+$ and $\tilde{G}^-$ are the two shifted components. For each pair of interfered modes with different line spacing and orientation, three images would be recorded with three different phases. Using matrix format, it can be expressed by:

$$\begin{bmatrix}\tilde{I}_1\\ \tilde{I}_2\\ \tilde{I}_3\end{bmatrix} = \begin{bmatrix}1 & 0.5e^{i\Delta\varphi_1} & 0.5e^{-i\Delta\varphi_1}\\ 1 & 0.5e^{i\Delta\varphi_2} & 0.5e^{-i\Delta\varphi_2}\\ 1 & 0.5e^{i\Delta\varphi_3} & 0.5e^{-i\Delta\varphi_3}\end{bmatrix} \times \begin{bmatrix}\tilde{O}^0\\ \tilde{G}^+\\ \tilde{G}^-\end{bmatrix} \qquad S\ 13$$

2. The reconstruction process of TVSFS labeled imaging

(a) Determination of the SFS vector

The zero-order component can be separated from the shifted components by multiplying an inverse matrix to Equation *S 13*.[2]

$$\begin{bmatrix}R_1\\ R_2\\ R_3\end{bmatrix} = \begin{bmatrix}1 & 0.5 & 0.5\\ 1 & 0.5e^{i\frac{2\pi}{3}} & 0.5e^{-i\frac{2\pi}{3}}\\ 1 & 0.5e^{i\frac{4\pi}{3}} & 0.5e^{-i\frac{4\pi}{3}}\end{bmatrix}^{-1} \begin{bmatrix}\tilde{I}_1\\ \tilde{I}_2\\ \tilde{I}_3\end{bmatrix}$$

$$= \begin{bmatrix}1 & 0.5 & 0.5\\ 1 & 0.5e^{i\frac{2\pi}{3}} & 0.5e^{-i\frac{2\pi}{3}}\\ 1 & 0.5e^{i\frac{4\pi}{3}} & 0.5e^{-i\frac{4\pi}{3}}\end{bmatrix}^{-1} \times \begin{bmatrix}1 & 0.5e^{i\Delta\varphi_1} & 0.5e^{-i\Delta\varphi_1}\\ 1 & 0.5e^{i\Delta\varphi_2} & 0.5e^{-i\Delta\varphi_2}\\ 1 & 0.5e^{i\Delta\varphi_3} & 0.5e^{-i\Delta\varphi_3}\end{bmatrix} \times \begin{bmatrix}\tilde{O}^0\\ \tilde{G}^+\\ \tilde{G}^-\end{bmatrix} \qquad S\ 14$$

$$= \begin{bmatrix}\tilde{O}^0 + c_0 \cdot \tilde{G}^+ + conj(c_0) \cdot \tilde{G}^-\\ r_1e^{i\phi_1} \cdot \tilde{G}^+ + r_2e^{i\phi_2} \cdot \tilde{G}^-\\ r_2e^{-i\phi_2} \cdot \tilde{G}^+ + r_1e^{-i\phi_1} \cdot \tilde{G}^-\end{bmatrix}$$

where c_0, r_1, r_2, ϕ_1 and $\phi_2$are constants. The zero-order component is separated out in R_1 in Equation *S 14*. The vector $\Delta\vec{K}$ can be determined by the peak location of correlation result between $\tilde{R}_2$ and $\tilde{G}^0 = \tilde{M} * \tilde{O} \cdot OTF \approx \tilde{M} * \tilde{O}^0$. With the SFS vector determined, phase $\Delta\varphi$ can be

calculated using the inverse matrix phase estimation algorithm (which is introduced in the followed part).

(b) Determination of the phase of the illumination pattern

To determine the three phases $\Delta\varphi_i$, four inverse matrixes in the form shown in Equation *S 15* is multiplied to Equation *S 13* (The details have been well presented in Ref [2].), where σ_i is positive real numbers and γ_i is real numbers.

$$\begin{bmatrix} \sigma_1 & 0.5e^{i\gamma_1} & 0.5e^{-i\gamma_1} \\ \sigma_2 & 0.5e^{i\gamma_2} & 0.5e^{-i\gamma_2} \\ \sigma_3 & 0.5e^{i\gamma_3} & 0.5e^{-i\gamma_3} \end{bmatrix}^{-1} \qquad S\ 15$$

Each multiplication with an inverse matrix can establish an equation of $\Delta\varphi_i$ by extracting the phase of correlation peak between updated R_2 and $\tilde{G}^0$. For an easier solving process, we established four equations of $\Delta\varphi_i$ to solve the three unknown $\Delta\varphi_i$.

(c) Calculation of the shifted components of the object.

$$\begin{bmatrix} \tilde{O}^0 \\ \tilde{G}^+ \\ \tilde{G}^- \end{bmatrix} = \begin{bmatrix} 1 & 0.5e^{i\Delta\varphi_1} & 0.5e^{-i\Delta\varphi_1} \\ 1 & 0.5e^{i\Delta\varphi_2} & 0.5e^{-i\Delta\varphi_2} \\ 1 & 0.5e^{i\Delta\varphi_3} & 0.5e^{-i\Delta\varphi_3} \end{bmatrix}^{-1} \times \begin{bmatrix} \tilde{I}_1 \\ \tilde{I}_2 \\ \tilde{I}_3 \end{bmatrix} \qquad S\ 16$$

With $\Delta\varphi_i$ solved, $\tilde{G}^+$and $\tilde{G}^-$can be calculated by:

$$\tilde{O}^{\pm} = \mathcal{F}\left[\frac{\mathcal{F}^{-1}\left(\tilde{G}^{\pm}\right)}{\mathcal{F}^{-1}\left(\tilde{M}\right)}\right] = \mathcal{F}\left[\frac{\mathcal{F}^{-1}\left(\tilde{G}^{\pm}\right)}{\frac{2A_1(\vec{r})A_2(\vec{r})}{[A_1(\vec{r})^2 + A_2(\vec{r})^2]}}\right] \qquad S\ 17$$

Then $\tilde{O}^+$ and $\tilde{O}^-$ can be calculated by:

where $\mathcal{F}^{-1}$ represents the inverse Fourier transform operator.

(d) Summing the recovered components and making apodization

With all calculated shifted components $\tilde{O}^{\pm}$, the complete Fourier spectrum of the object can be achieved by shifting them back to their correct position in the spatial frequency domain and

summing them together. Fourier transforms of the calculated frequency spectrum would give a super-resolution image but with severe artifact due to the unequal weight in the overlapped region. So we deconvolve the reconstructed image with the calculated PSF to equalize the weights in the Fourier domain.[3]

For labeled imaging, we use the incoherent imaging model, so that

$$k_c = \frac{2NA}{\lambda_{em}} \quad S\ 18$$

$$k_s = \frac{2n_{PC} \cdot sin\theta}{\lambda_{ex}} \quad S\ 19$$

By adopting Equation 4, we can deduce the resolution of

$$\Delta_{xy} = \frac{\lambda_{em}}{2\left(NA + \frac{n_{PC} \cdot sin\theta \cdot \lambda_{em}}{\lambda_{ex}}\right)} \quad S\ 20$$

Supplementary Note 3: Error analysis of the chip design.

The location of the illuminated evanescent waves is key important for the successful implementation of the chip-based super-resolution imaging. To analyze the location error of the illumination, we need

$$n_{PC} \cdot sin\theta = m \cdot \frac{\lambda_{ex}}{p} \ (m \text{ is the integral number}) \qquad S\ 21$$

to start from the grating equation:

where n_{PC} represents the refractive index of the GaP crystal at the wavelength of λ_{ex}, θ is the diffractive angle in the GaP crystal and p is the period of the grating. The values of m specify the order of the various principal maxima. Here we only use the 1st order of the diffraction light for the comparatively high diffraction efficiency. The 0th and higher order (2nd,3rd…) diffraction will be blocked out by the metal film evaporated on the upper surface of the GaP chip.

The distance (r) between the grating and the illuminated evanescent waves depends on the

$$r = T \cdot tan\theta \qquad S\ 22$$

thickness (T) and the angle of the first order diffraction of the GaP crystal:

$$r = T \cdot \frac{\lambda_{ex}}{\sqrt{(p \cdot n_{PC})^2 - \lambda_{ex}{}^2}} \qquad S\ 23$$

Substitute (1) into (2), the distance becomes:

$$\frac{dr}{dp} = \frac{-T \cdot \lambda_{ex} \cdot p \cdot n_{PC}{}^2}{\left((p \cdot n_{PC})^2 - \lambda_{ex}{}^2\right)^{\frac{3}{2}}} \qquad S\ 24$$

The partial derivative of r with respective to p can be expressed as:

$$\frac{dr}{dT} = \frac{\lambda_{ex}}{\sqrt{(p \cdot n_{PC})^2 - \lambda_{ex}{}^2}} \qquad S\ 25$$

The partial derivative of r with respective to T can be expressed as:

As can be seen from Equation *S 24* and Equation *S 25*, the precision of grating period is vital

important for the correct location, especially when the grating period is small (see **Figure S9**).

Supplementary Note 4: Determining the FOV of TVSFS method.

The FOV is determined by both the chip illumination region and the imaging system. Here we only consider the chip illumination region since the chip illumination is more crucial in the super-resolution reconstruction and the FOV can be easily extended by moving the objective lens. The FOV of the chip illumination is ideally the overlap between evanescent waves coupled from various gratings. Considering square gratings with side length of *d*, the shape of light transported onto the sample is a square with a side length of *d*, as indicated in the **Figure S11**, where *m* is the number of gratings per SFS magnitude, *N* is the side number of the overlapped region. The area of

$$\frac{N}{4} tan \frac{180°}{N} \cdot d^2 \qquad S\ 26$$

FOV is related to *N* as:

When the side number *N* approaches infinite, the FOV is approximate to a circle with a radius of $\frac{d}{2}$, thus the area of FOV is $lim_{N \to \infty} \frac{N}{4} tan \frac{180°}{N} \cdot d^2 = \pi \frac{d^2}{4}$.

Supplementary Note 5: Performance of TVSFS imaging with photon noise.

In real imaging systems, photon noise, also known as Poisson noise, constitutes the dominant source of image noise in most cases. The signal to noise ratio (SNR) of the raw image can be related with

$$SNR = \sqrt{N} \qquad S\,27$$

the photon number (N) as:

We apply Poisson noise into the raw images to simulate the performance of the TVSFS method under illumination with different levels of photon number, as shown in **Figure S12** and **Figure S13**. For each simulation, the peak photon number (PPN) of the raw images is set to be 10,100,1000 and 10000 before Poisson noise is applied. Furthermore, the line scans of the structures with distance around the resolution limit are compared. For both labeled and label-free imaging, when the maximum photon number reaches 1000, the reconstruction is close to the case without noise, which can be judged from the real and Fourier spaces. For label-free imaging, the double line with 125 nm (around the theoretical resolution limit) cannot be dissolved using the Rayleigh criterion when the PPN is 10 and 100, and the Fourier space shows some unsmooth connection. For labeled imaging, the Fourier space shows disconnections under the low signal of 10 and 100, caused by the wrong estimation of the phase and wavevector. The flaw in the Fourier space also leads to poor performance in the real space, especially when the structure is around the theoretical resolution limit (~80 nm).

Supplementary Note 6: Imaging speed of TVSFS method.

A comparison between the TVSFS method and point-scanning super-resolution imaging method:

We only discuss the acquisition time since the reconstruction time can be reduced using a more advanced computer and optimization algorithm. For example, for commercial STED, the dwell time for a pixel (50 nm) is 20 μs, then for a FOV of 20 μm × 20 μm, we can calculate the imaging time to be 3.2 s/frame.

For the TVSFS method, to get 50 nm resolution, a SiC substrate can be used (see **Table S1**). A full spectrum requires 24 (4 directions × 3 phases × 2 SFSs) raw images, as shown in the spectrum in **Figure S14**. Suppose an acquisition time of 20 ms is required for every raw image, then the imaging time can be calculated to be 480 ms/frame, which is much faster than commercial STED microscopy. Besides, the scheme of wide-field acquisition makes this advantage more apparent when a FOV is bigger than 20 μm × 20 μm imaged.

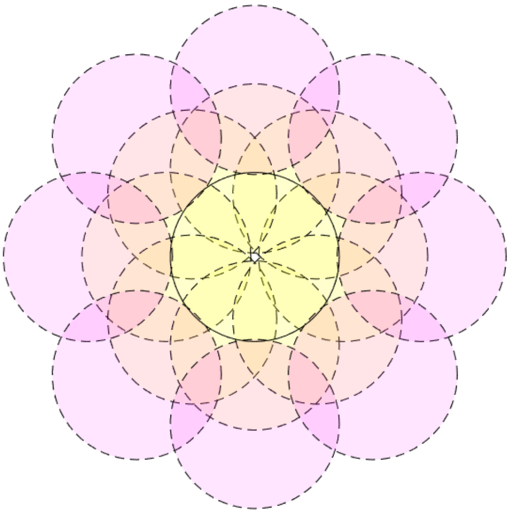

Figure S14. Simulation parameters: NA: 1.49, λ_{ex}: 405 nm, λ_{ex}: 421 nm, SFS magnitudes: 3.0 k_0, 5.6 k_0, which corresponds to a resolution of 48 nm.

The imaging speed of the TVSFS method can be further improved using the deep-learning method based on two points:

Firstly, it has been demonstrated that the deep-learning-based super-resolution method shows less residual difference than conventional super-resolution images under imaging conditions of low

fluorescence because data-driven deep-learning-based super-resolution approaches are usually conducive to separating noise from biological structures.[4, 5] Based on this principle, the imaging speed can be improved by decreasing the exposure time of every raw image.

Secondly, the imaging speed can also be improved by reducing the number of raw images with decreasing overlapping rates in the spectrum.[6]

Overall, the deep-learning-based method can significantly improve the imaging throughput by reducing acquisition and computational times. Once the deep network is trained, it remains fixed and can be used to rapidly output batches of high-resolution images using a graphics processing unit (GPU).

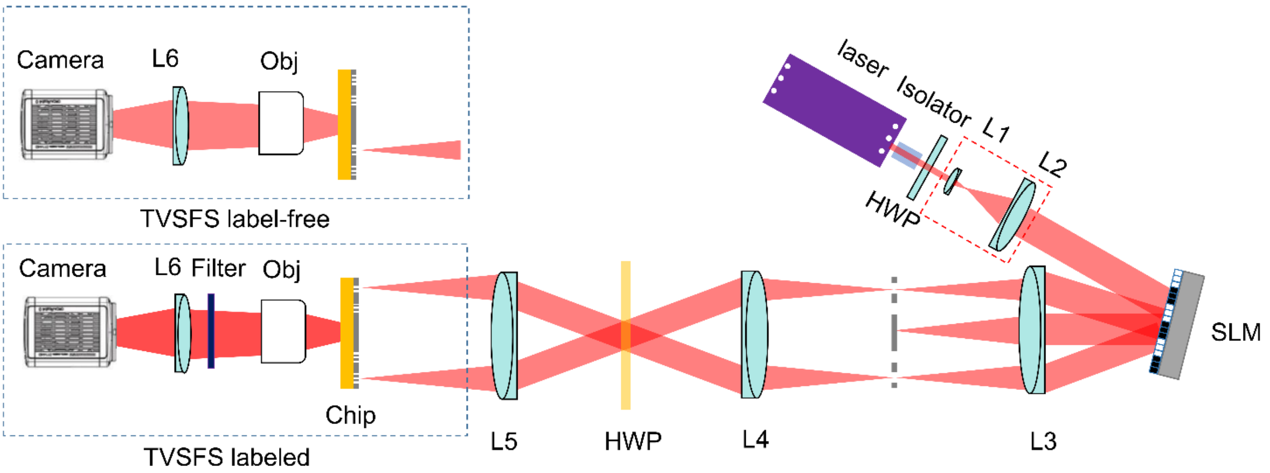

Figure S1. Schematic of chip-based TVSFS imaging. Optomechanical configuration of the microscope. L1-L8: optical lens; HWP: Half wavelength plate; SLM: spatial light modulator; Obj: objective lens; The optical path was compatible with both labeled and label-free TVSFS imaging by uploading different binary images at the SLM. For labeled TVSFS imaging, binary grating patterns were used to generate double beams for illumination. For label-free TVSFS imaging, we used blazed grating patterns to only allow one beam passing through the chip. The zero-order light has been blocked out by the thick metal film evaporated at the center of grating patterns on the chip.

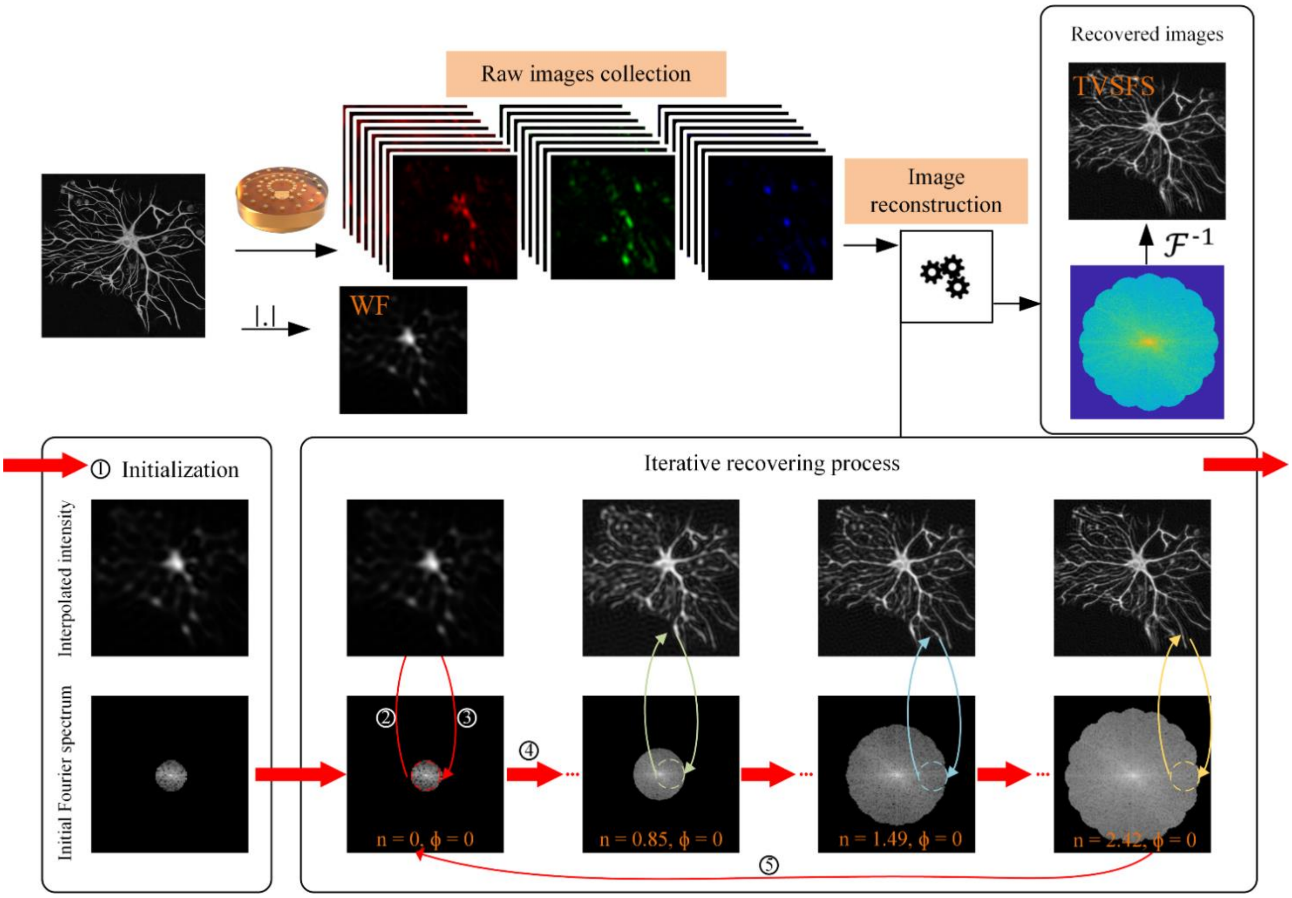

Figure S2. Iterative recovery procedure of TVSFS label-free imaging. The reconstruction algorithm is illustrated in 5 steps. Step 1: initialize the high-resolution image, $\sqrt{I_h}e^{i\varphi_h}$. Step 2: low filter the in the Fourier space and inverse Fourier transform to generate the low-resolution image $\sqrt{I_l}e^{i\varphi_l}$. Step 3: replace the intensity $\sqrt{I_l}$ with the intensity measurement $I_{k_s,\phi}$, that is $\sqrt{I_{k_s,\phi}}e^{i\varphi_l}$ and update in the Fourier space. Step 4: repeat steps 2–3 for other plane-wave incidences (total of $N(k_s) \cdot N(\phi)$ intensity images). Step 5: repeat steps 2–4 until the convergence.

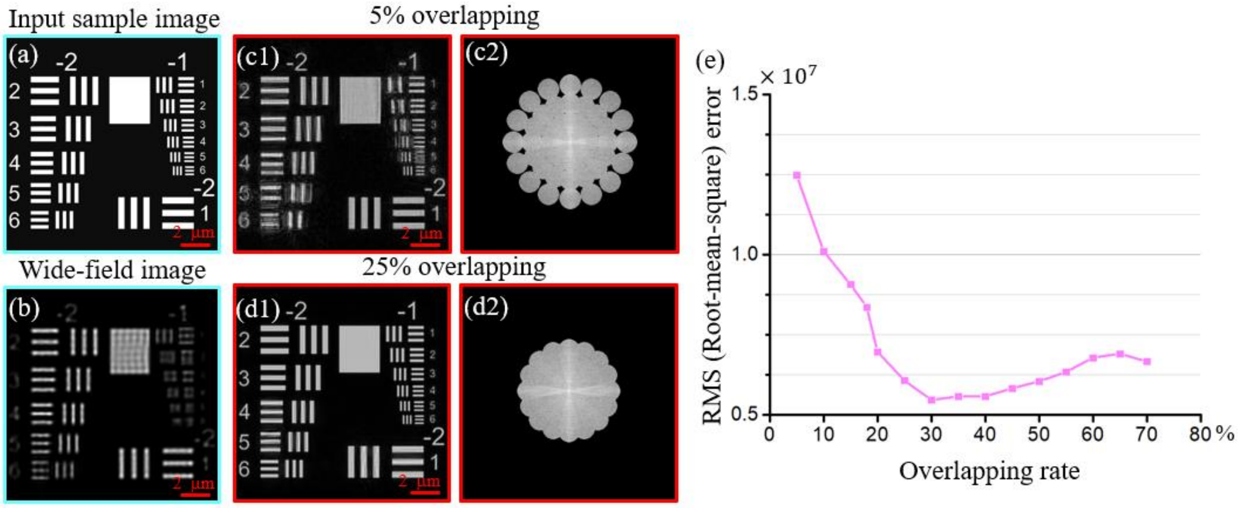

Figure S3. Reconstructions of TVSFS label-free imaging with different spectrum overlapping percentages. (a) The input high-resolution intensity of the simulated sample. (b) The wide-field image of simulated sample. (c-d) TVSFS label-free reconstructions and the spectrum with 5% and 25% overlapping percentages in the Fourier domain. (e) The RMS (Root-mean-square) error of TVSFS label-free reconstructions with different spectrum overlapping percentages.

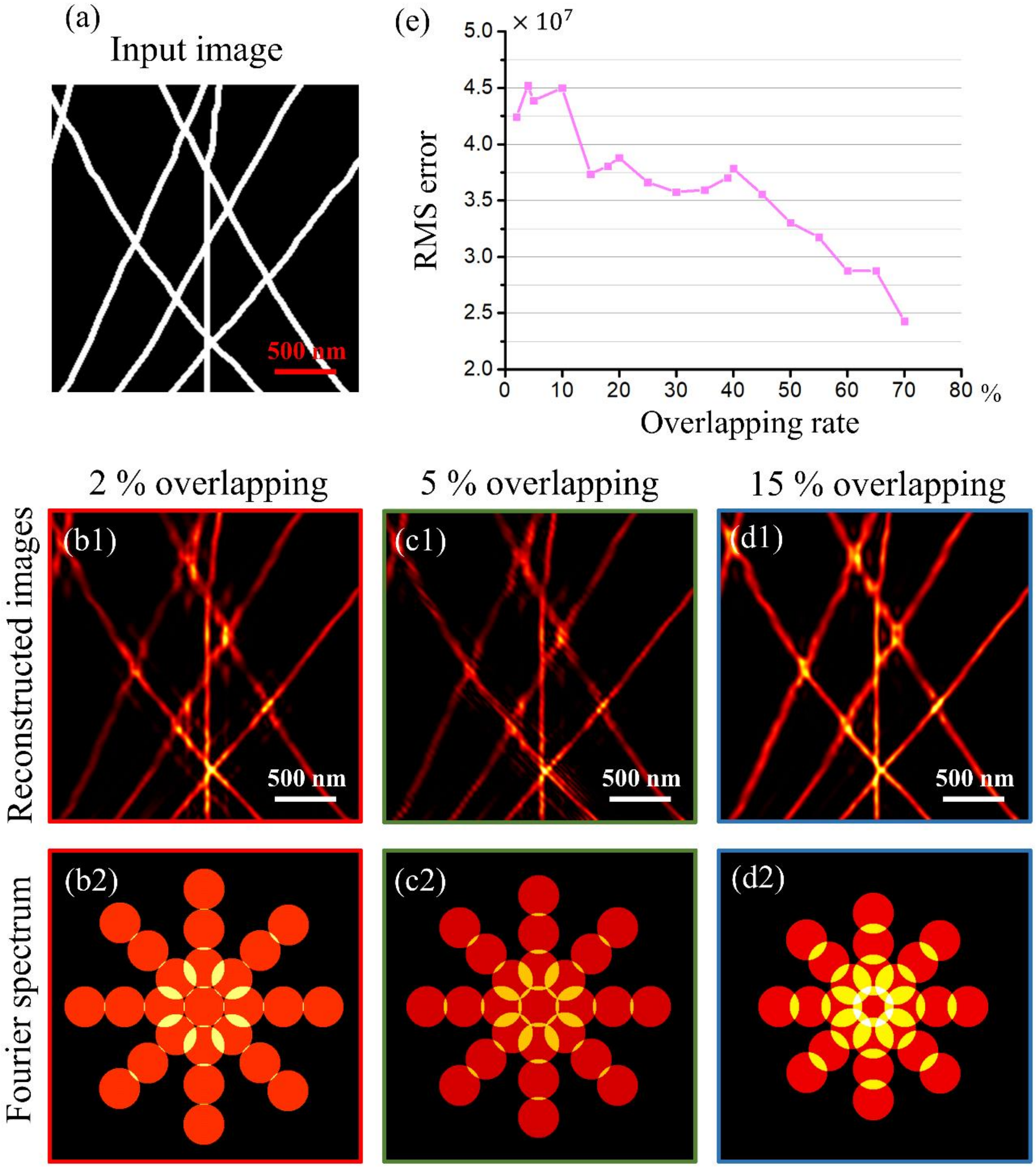

Figure S4. Reconstructions of TVSFS labeled imaging with different spectrum overlapping percentages. (a) The input high-resolution intensity of the simulated sample. (b) The RMS (Root-mean-square) error of TVSFS labeled reconstructions with different spectrum overlapping percentages. (c-e) TVSFS labeled reconstructions with 2%,5%,15% overlapping percentages in the Fourier domain.

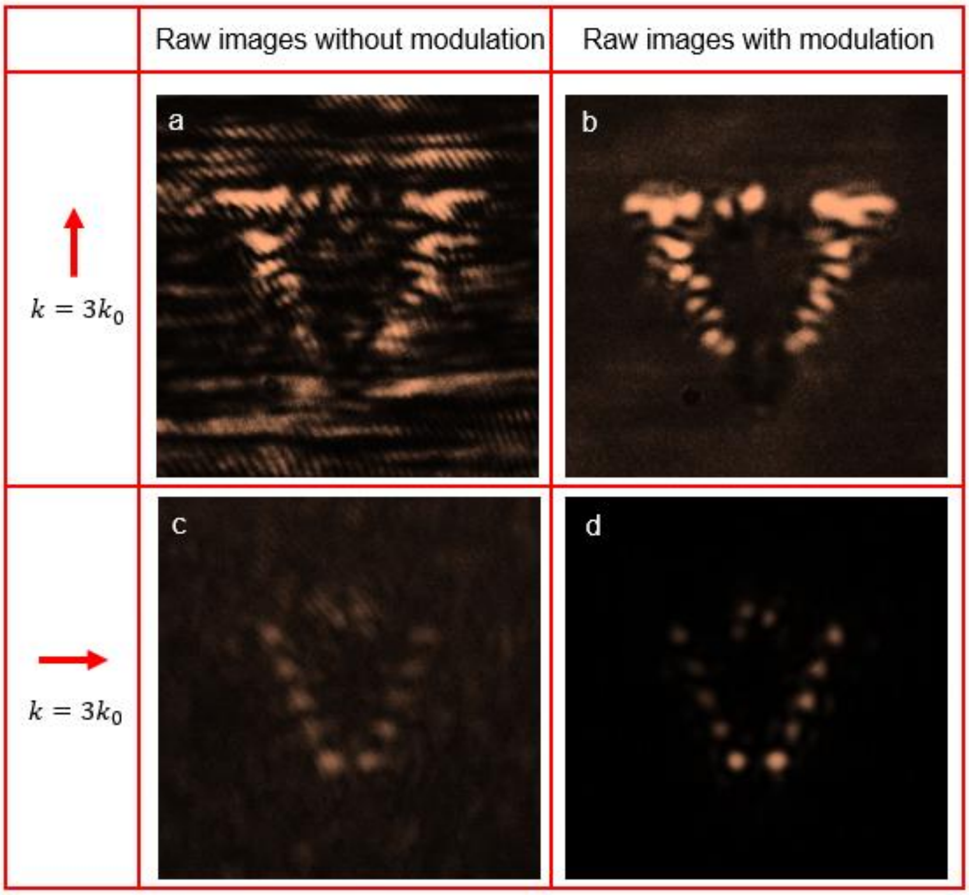

Figure S5. Comparison of TVSFS label-free raw images with or without laser modulation. The 660 nm laser diode was modulated by applying a sawtooth wave (frequency:100Hz amplitude:100 mV) with a function generator. (a, c) TVSFS label-free raw images without laser modulation. (b, d) TVSFS label-free raw images with laser modulation. The red arrows indicate the illumination directions and corresponding SFS magnitudes.

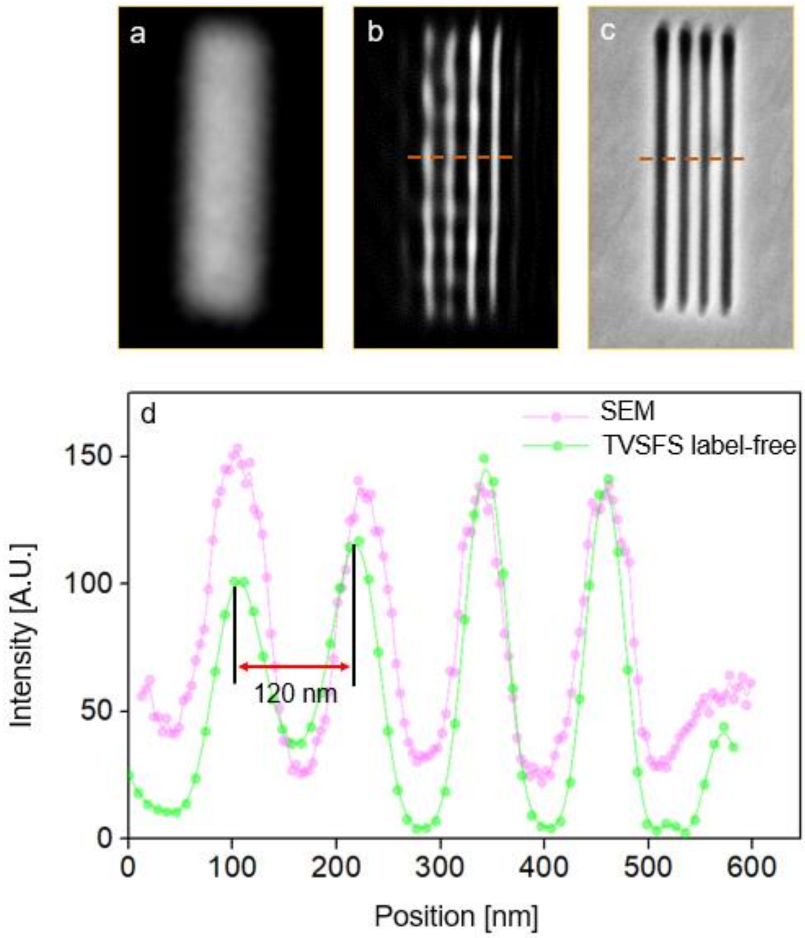

Figure S6. Resolution calibration of TVSFS label-free imaging using the 561 nm laser and 1.49-NA objective. (a-c) The images of a four-line-slits taken under wide field, TVSFS label-free, and SEM respectively. The four-line-slits was fabricated using FIB and has a 120-nm center to center distance. (d) The line comparison between (b) and (c) shows a good correspondence between TVSFS label-free and SEM, which could not be achieved by the wide field.

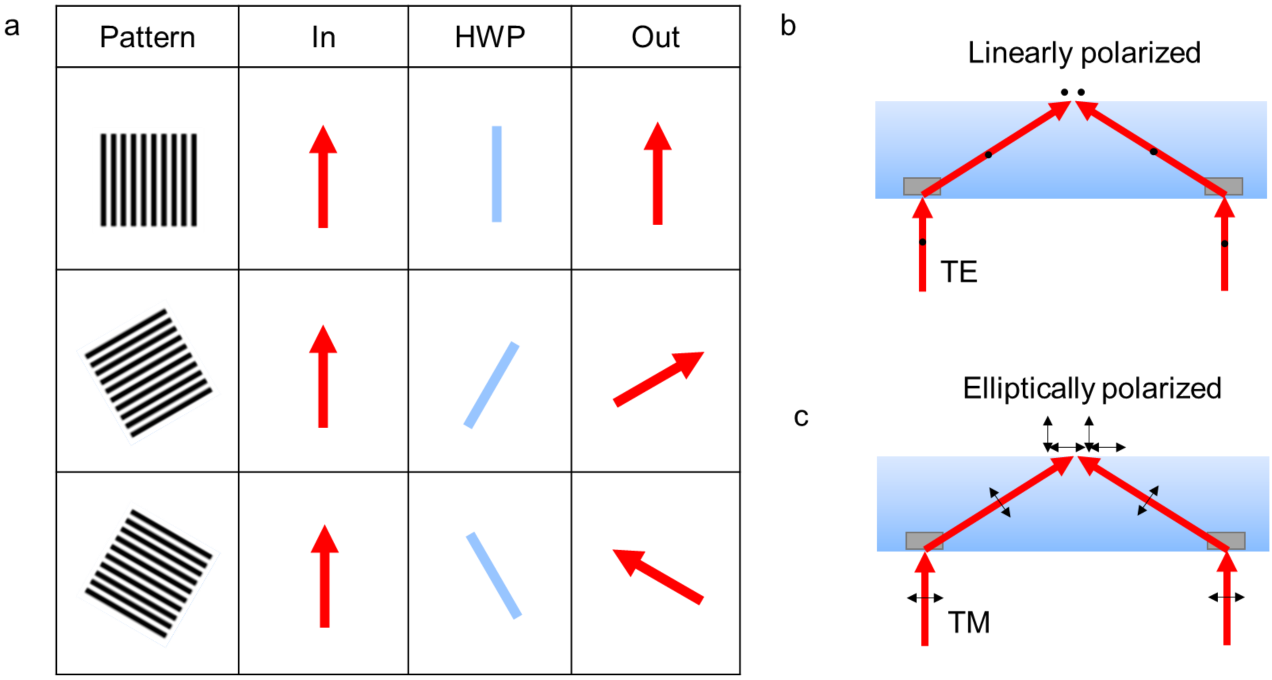

Figure S7. Polarization control in TVSFS label imaging. (a) Different patterns generated by the SLM are coupled with the corresponding output polarizations with half wavelength plate (HWP). First column: three patterns with different orientations generated by the SLM; second column: the same illumination polarizations before entering the HWP; third column: the corresponding changes in polarization induced by the HWP. fourth column: light refracted from grids of different orientations displays different polarizations after passing through the HWP. (b-c) The principle of polarization control to maximize the interference contrast on the photonic chip. The incident of TE polarization light will keep the polarization direction and forms the linearly polarization on the surface of the photonic chip, as indicated in (b). The incident of TM polarization light will change the polarization direction after diffraction and forms the elliptically polarization on the surface of the photonic chip, as indicated in (c). The interference of elliptically polarized light will have a decreased contrast compared with linearly polarized light.

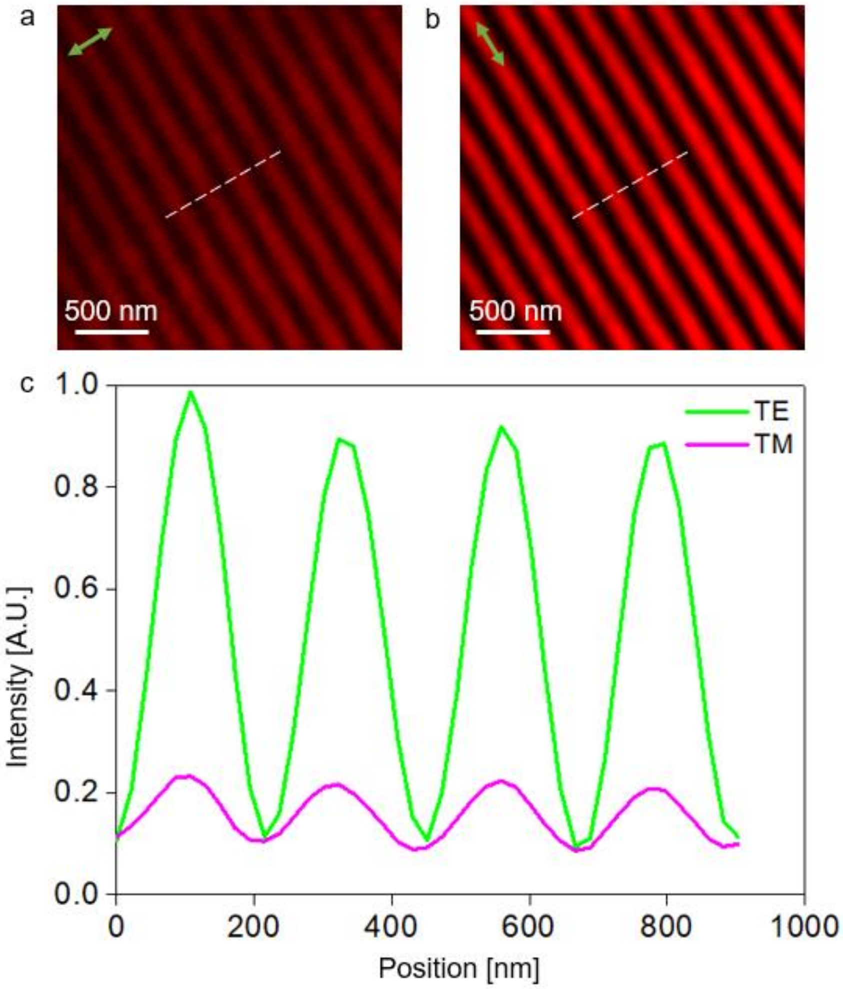

Figure S8. Polarization control in TVSFS labeled imaging. (a-b) The evanescent interference patterns generated by the TE (a), and TM light (b). (c) Line profiles, taken at the position indicated with a white dash line in a, b, showing that evanescent wave generated by TE light has a better contrast and light intensity compared with TM light.

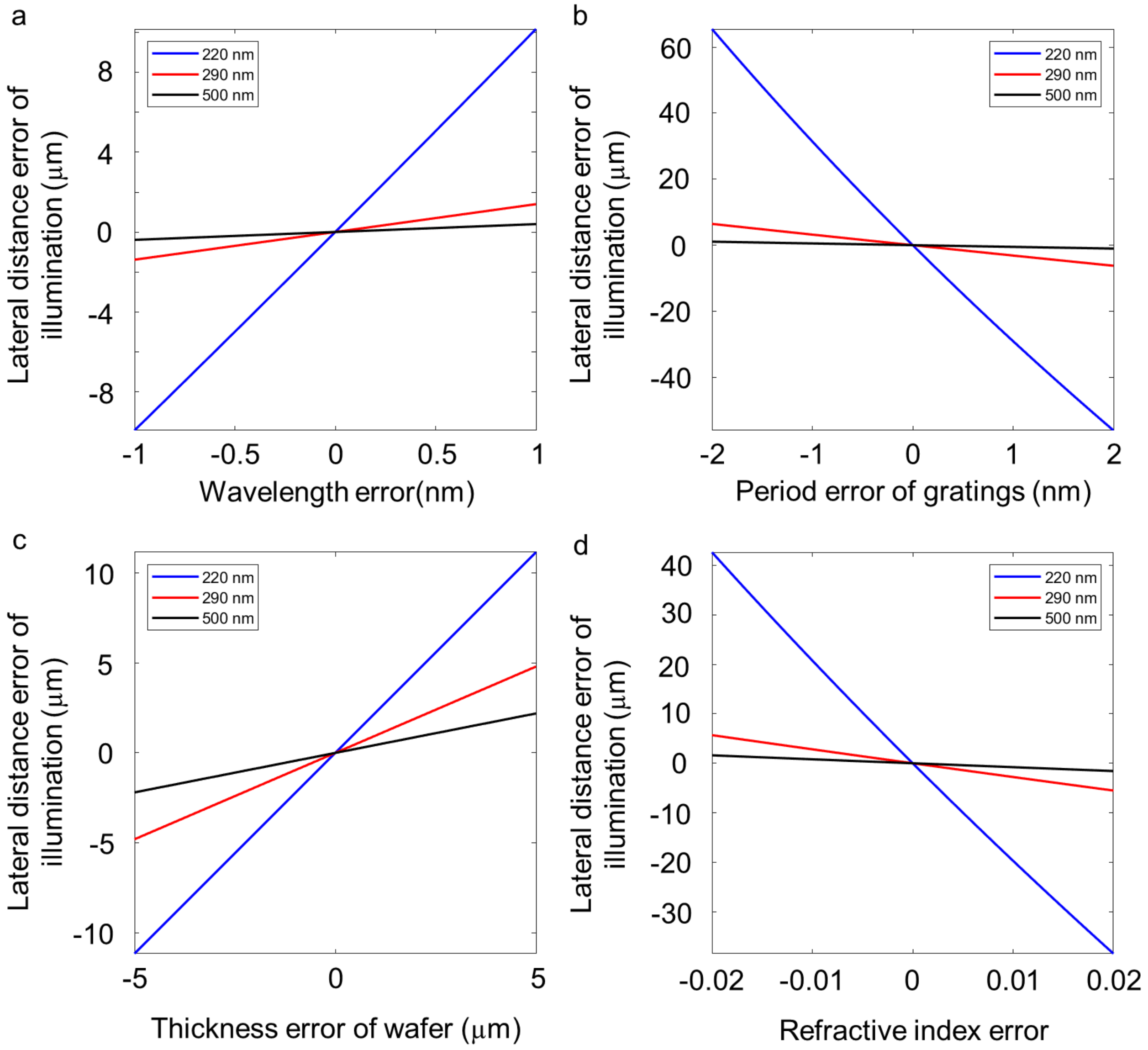

Figure S9. Error analysis of the chip fabrication.

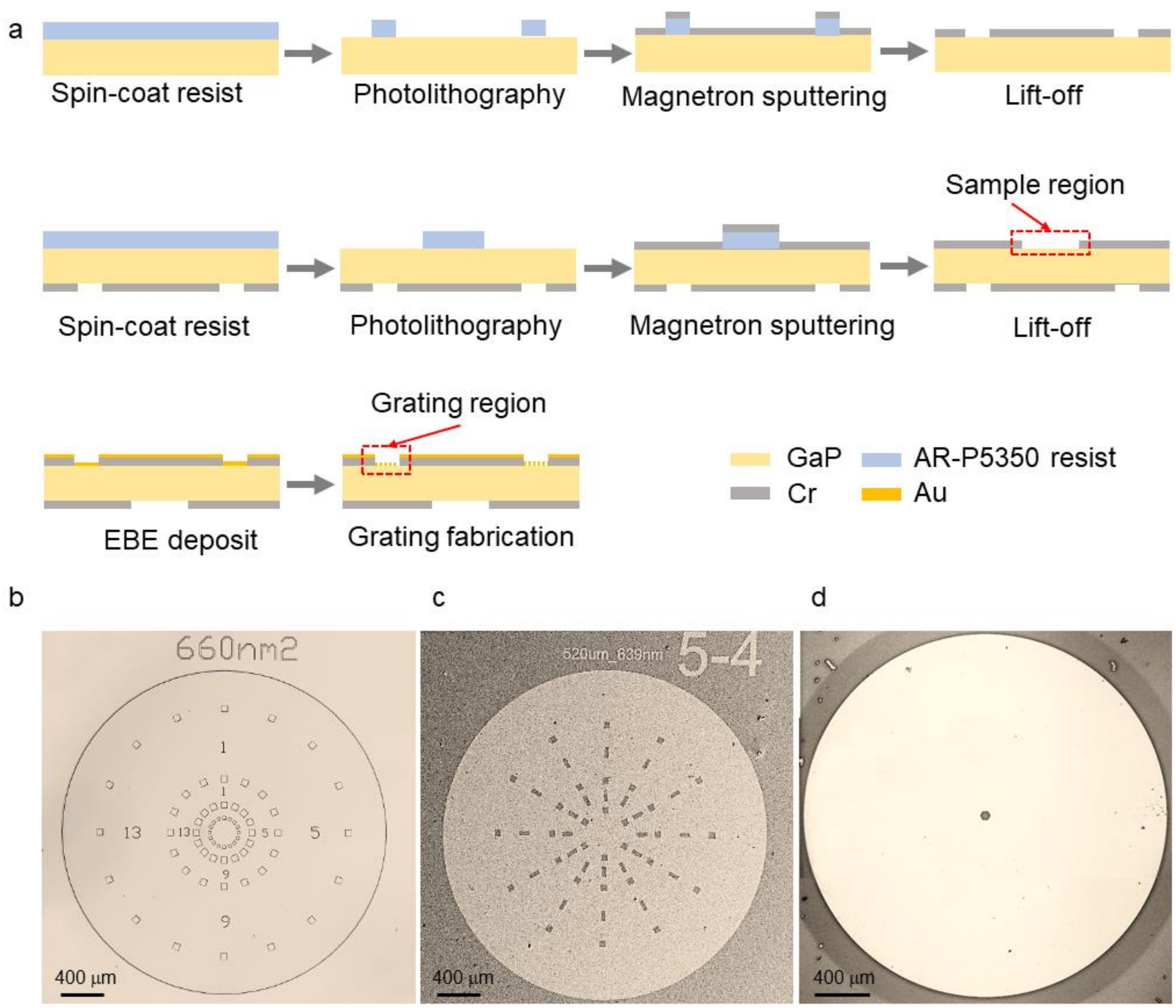

Figure S10. Modified chip design for TVSFS imaging with more light blocking. (a) The fabrication process of the TVSFS chip. (b-c) The grating-side images of two fabricated TVSFS chips with different parameters. (d) The sample-side image of the fabricated TVSFS chips, with space for putting the sample while the other places are covered with Cr film.

a

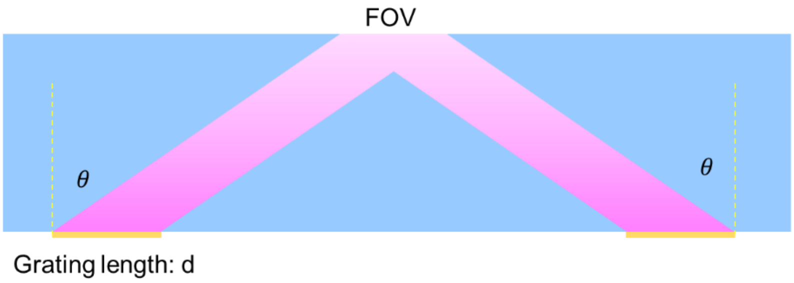

b

m	4	8	6,12	∞
Schematic figure	d 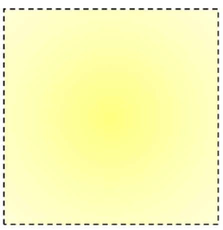	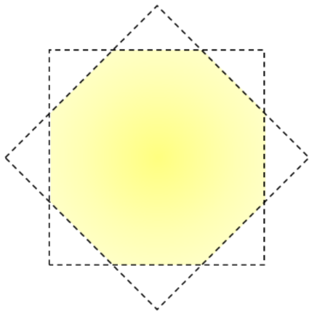	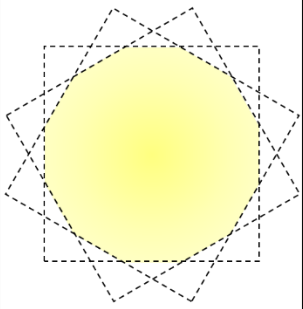	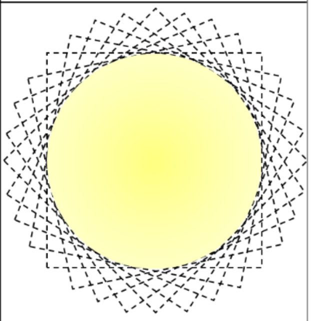
N	4	8	12	∞
FOV	d^2	$\frac{2\sqrt{2}}{2+\sqrt{2}}d^2$	$\frac{3}{2+\sqrt{3}}d^2$	$\lim_{N\to\infty}\frac{N}{4}tan\frac{180°}{N}\cdot d^2=\pi\frac{d^2}{4}$

Figure S11. FOV determination for TVSFS imaging.

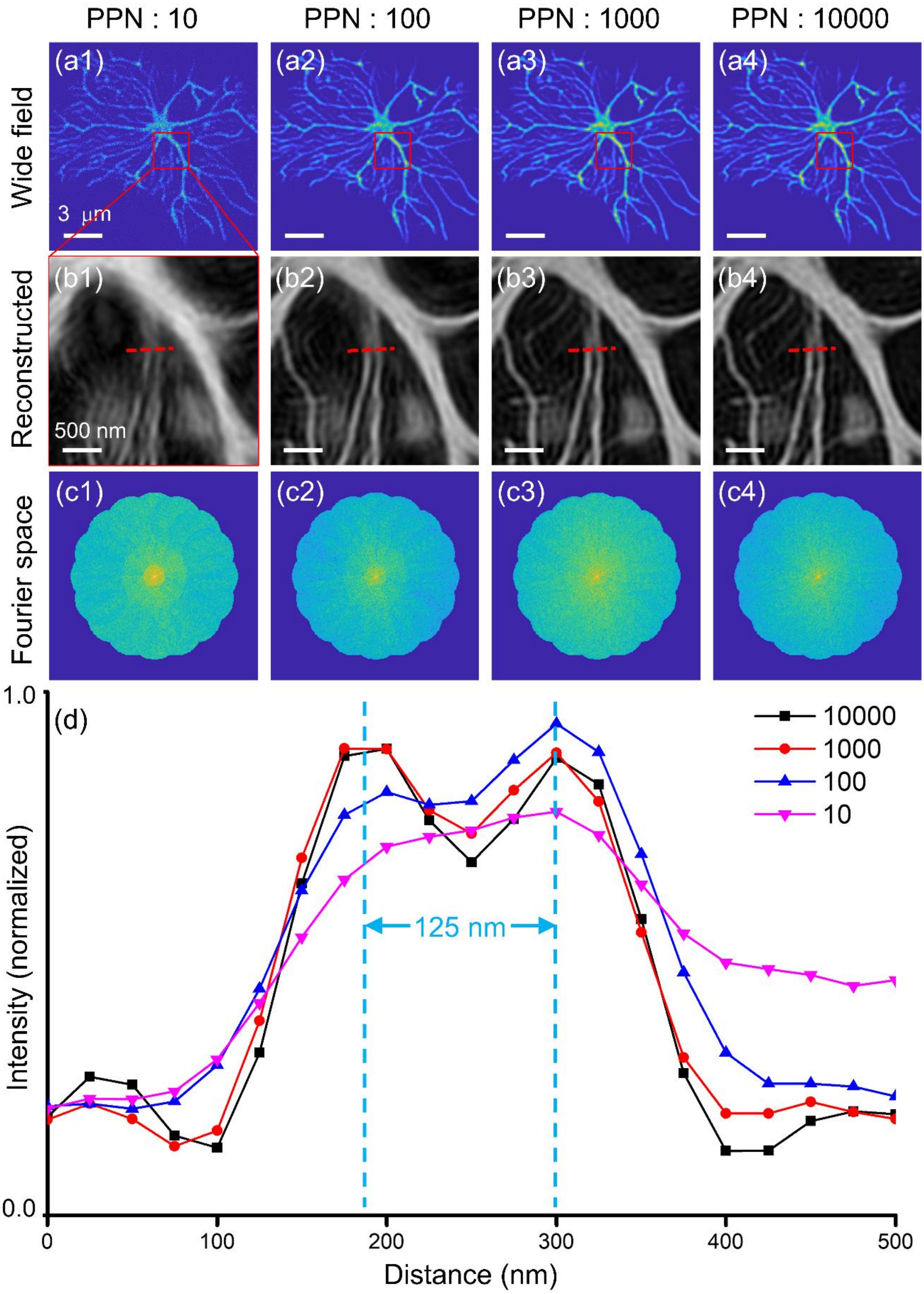

Figure S12. Performance of label-free imaging with Poisson noise. Simulation with NA of 1.49, the wavelength of 561 nm, and SFS magnitudes of $1.0k_0$, $2.1k_0$, $3.3k_0$. The peak photon numbers (PPN) of 10,100,1000, and 10000 are set before Poisson noise is applied. (a1-a4) Wide-field image of an astrocyte with different PPN. (b1-b4) TVSFS image of the inset in (a1-a4). (c1-c4) Fourier space of the TVSFS images. (d) The line comparison of (b1-b4).

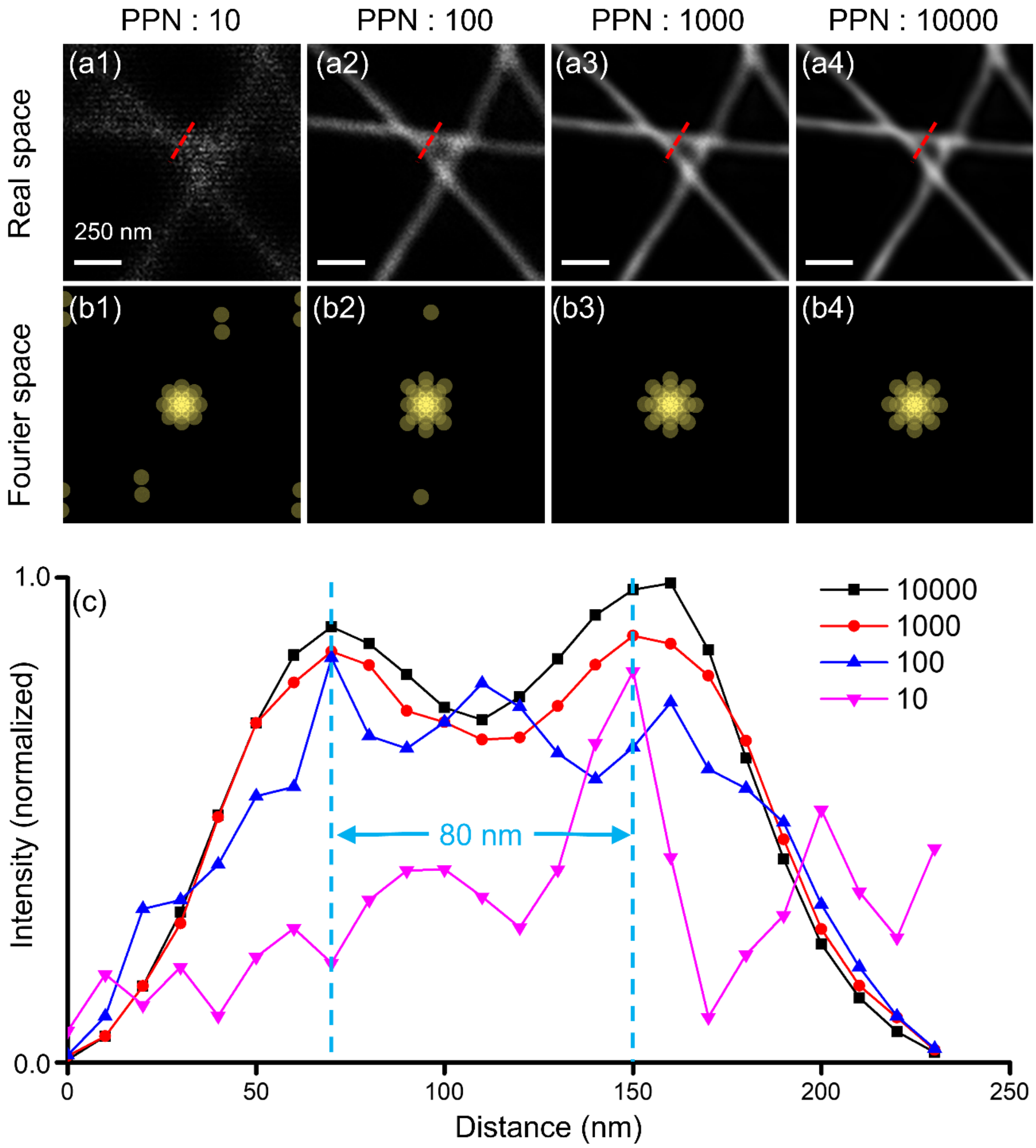

Figure S13. Performance of labeled imaging with Poisson noise. Simulation with NA of 1.1, excitation wavelength of 639 nm, an emission wavelength of 661 nm, and SFS magnitudes of $1.8k_0$, $3.2k_0$, $4.8k_0$ and $6.6k_0$. The peak photon numbers (PPN) of 10,100,1000, and 10000 are set before Poisson noise is applied. (a1-a4) TVSFS image of a microtubule pattern with different PPN. (b1-b4) Fourier space of the TVSFS images. (c) The line comparison of (a1-a4).

Table S1. The refractive index, extinction coefficient of GaP[7] and 4H-SiC,[8] and the ultimate resolution for various wavelengths with NA of 1.49. The resolution using GaP substrate when the excitation wavelength is less than 560 nm is not shown for the large optical absorption.

λ_{ex}	λ_{em}	GaP		Resolution (nm)		SiC		Resolution (nm)	
(nm)	(nm)	n	k	Label-free	Labeled	n	k	Label-free	Labeled
405	421	4.1522	0.2556	-	-	2.8285	0	94	48
488	519	3.6408	0.0081	-	-	2.7489	0	115	59
532	553	3.4932	0.0026	-	-	2.7233	0	126	64
561	603	3.4266	0.0002	114	58	2.7101	0	134	68
639	665	3.3112	0	133	67	2.6838	0	153	78
660	690	3.2882	0	138	70	2.6783	0	158	80

References:

[1] G. Zheng, R. Horstmeyer, C. Yang, *Nat. Photonics* **2013**, 7, 739.

[2] R. Cao, Y. Chen, W. Liu, D. Zhu, C. Kuang, Y. Xu, X. Liu, *Biomed. Opt. Express* **2018**, 9, 5037.

[3] V. Perez, B.-J. Chang, E. H. K. Stelzer, *Sci. Rep.* **2016**, 6, 37149.

[4] M. Weigert, U. Schmidt, T. Boothe, A. Muller, A. Dibrov, A. Jain, B. Wilhelm, D. Schmidt, C. Broaddus, S. Culley, M. Rocha-Martins, F. Segovia-Miranda, C. Norden, R. Henriques, M. Zerial, M. Solimena, J. Rink, P. Tomancak, L. Royer, F. Jug, E. W. Myers, *Nat. Methods* **2018**, 15, 1090.

[5] C. Qiao, D. Li, Y. Guo, C. Liu, T. Jiang, Q. Dai, D. Li, *Nat. Methods* **2021**, 18, 194.

[6] T. Nguyen, Y. Xue, Y. Li, L. Tian, G. Nehmetallah, *Opt. Exp.* **2018**, 26, 26470.

[7] D. E. Aspnes, A. A. Studna, *Phys. Rev. B* **1983**, 27, 985.

[8] S. Wang, M. Zhan, G. Wang, H. Xuan, W. Zhang, C. Liu, C. Xu, Y. Liu, Z. Wei, X. Chen, *Laser Photon. Rev.* **2013**, 7, 831.
